# Concentration Dependent Effect of Quaternary Amines on the Adhesion of U251-MG Cells

**DOI:** 10.3390/gels8120827

**Published:** 2022-12-15

**Authors:** Nils Stamm, Kristin Glotzbach, Andreas Faissner, Ralf Weberskirch

**Affiliations:** 1 Faculty of Chemistry and Chemical Biology, TU Dortmund University, 44227 Dortmund, Germany; 2 Department of Cell Morphology and Molecular Neurobiology, Ruhr Universität Bochum, 44801 Bochum, Germany

**Keywords:** cationic hydrogels, U251-MG cells, cell adhesion, live/dead assay

## Abstract

Cationic gels have seen increasing interest in recent years for 2D cell cultivation since they may represent an alternative to the well-known RGD-peptide motif functionalized gels. However, few hydrogel systems with adjustable cationic strength have been fabricated and investigated so far. In this work, eight gels with defined concentrations of cationic groups, two of which also contained the RGD peptide, were prepared from three well-defined, soluble precursor copolymers with thiol-functionalities and PEGDA3500 as a crosslinker via thiol-ene chemistry. Live/dead stainings of U-251-MG cells on the hydrogels with different concentrations of the cationic motif were made after 3 days and 7 days of cultivation. The results show a high dependence of the number of adhesive cells and their morphology, cluster versus spread cells, on the concentration of cationic groups in the gel. This effect was more pronounced when the gels were not further dialyzed before usage. In addition, a synergistic effect of the two motifs, cationic group and RGD peptide, could be demonstrated, which together induce stronger cell adhesion than either motif alone.

## 1. Introduction

Cell therapies play an increasingly important role in medicine for the treatment of multiple diseases [1,2]. However, the low rate of cell survival and uncontrolled differentiation remain major challenges of this approach. One class of materials that has found increasing attention in cell therapies is hydrogels. Hydrogels are physically or chemically crosslinked 3D polymer networks that absorb water and can be of natural or synthetic origin. Hydrogels based on natural raw material sources, such as proteins or polysaccharides, have to deal with problems of batch-to-batch variability in composition and it is sometimes unclear what biological function the individual components have [3]. On the other hand, hydrogels based on synthetic polymers can be produced reproducibly with defined composition, functionality, and stiffness. Consequently, hydrogels provide an ideal, physicochemical mimetic of native extracellular matrix (ECM) that can be utilized as a delivery vehicle for cells [4,5]. Moreover, hydrogels find increasing interest to culture cells in 2D or encapsulate them in 3D to study cellular behavior in conditions that resemble more the natural environment of cells [6,7,8]. To enable synthetic hydrogels to mediate cell adhesion, they are often functionalized with peptides, such as the arginine–glycine–aspartic acid (RGD) tripeptide from fibronectin [9]. This peptide interacts with various integrins on the cell surface such as α_v_β_3_ and α_5_β_1_ that are well-known fibronectin receptors [10,11,12] and thereby contribute significantly to cellular adhesion. A second approach to facilitate cell adhesion is based on cationic surfaces such as coated poly(L-lysine) due to its ability to promote cell adhesion in a nonspecific manner via electrostatic interactions with the negatively charged cell membrane [13,14,15]. Cationic charges are an interesting alternative to RGD peptides, as they are much easier to introduce into polymers. In contrast to RGD peptides, however, the mechanism of cell adhesion is based on non-specific, electrostatic interactions with the negatively charged pericellular matrix (PCM) composed of hyaluronan brushes and a positively charged substrate surface. After this initial cell-surface attachment, the formation of RGD integrin interactions can occur [16,17]. Despite these interesting developments occurring with cationic hydrogels, only a few studies investigated systematically the influence of cationic charge on cell adhesion. The majority of papers that use cationic polymers for 2D/3D cell adhesion use poly-L-lysine in combination with other polymers either grafted or as blends [18,19,20]. Such combinations of poly-L-lysine with other polymers such as PEG have often shown that by changing the cationic charge density also other parameters of the gels are changed such as mesh size, swelling behavior, or stiffness, which makes it more difficult to assign cellular behavior only to the change in cationic group concentration [21]. Other reports have shown that higher cationic concentration can induce cell lysis [22] and decreased viability of mouse fibroblast cells [23,24], whereas lower cationic concentration may not have any effect on cell adhesion at all. Therefore, application of cationic gels for cell culturing remains a challenging topic since the materials are very often not well-defined and cellular effects cannot easily be attributed to the cationic charge density alone, as other gel parameters often change as well. Therefore, well-defined synthetic materials with variable cationic group concentration should be helpful to better understand the effect of cationic charge density on cellular behavior. More recently, we have developed a hydrogel material where cationic group densities can be changed while keeping the stiffness and swelling behavior of the gel nearly constant [25].

In this study, we used the basic design of the fully synthetic hydrogel system based on 4-Acryloylmorpholine (AMor), *N*,*N*′-Bis(methacryloyl)cystamide (BMAC), Trimethylaminoethyl acrylate (TMAEA), and PEGDA3500 as crosslinker to generate fully analyzable and easily modifiable hydrogel systems. In this study, we investigated the concentration-dependent effect of a quaternary amine (TMAEA) with a permanent cationic charge on the adhesion and survival of U-251-MG cells on hydrogels and compared the cellular behavior to neutral gels and gels that contained cationic groups in combination with the well-known RGD-peptide motif.

## 2. Results and Discussion

### 2.1. Prepolymer Synthesis

**Figure 1:**
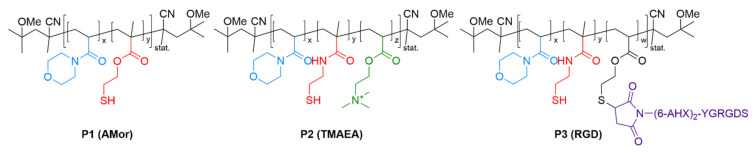
Polymers used for the synthesis of hydrogels. **P1 (AMor)** containing AMor (blue) as the hydrophilic group, BMAC (red) as a thiol containing monomer, and no further functionalization; **P2 (TMAEA)** containing additional TMAEA (green) as a permanent cationic group; and **P3 (RGD)** containing additional MIC-6AHX-YGRGDS peptide sequence (violet).

Many different parameters such as chemical and biological signals as well as mechanical properties of the material have a great influence on the cultivation of cells. For example, the adhesion of cells can be improved non-specifically via cationic charges, which can interact with the negatively charged membrane of cells [26], or specifically via the interaction of integrins with the well-known RGD peptide sequence [27]. To study different influences on cell development, a simple system that can be functionalized with different bioactive motifs is necessary. We present here a modifiable polymer system consisting of the basic components AMor (hydrophilic component) and BMAC (thiol-containing component), which can be further functionalized using additional monomers or thiol-ene chemistry. The hydrogels were prepared by thiol-ene Michael reaction from thiol-containing prepolymers and the crosslinker PEGDA3500. To investigate the influence of cationic groups and peptides on cell behavior during in vitro cultivation, three prepolymers were first synthesized. In Figure 1, the unfunctionalized polymer (P1) consisting of the hydrophilic component AMor (blue) and the thiol-containing component BMAC (red) as a reference, the cationic polymer (P2) with the additional quaternary amine-containing monomer TMAEA (green), and the peptide polymer (P3) with the additional peptide sequence MIC-6AHX-YGRGDS (Maleimidocaproyl-6-aminohexanoic acid- tyrosine-glycine-arginine-glycine-aspartic acid-serine) (violet) are shown.

The prepolymers were prepared by free radical polymerization and analyzed by proton nuclear magnetic resonance (^1^H-NMR) spectroscopy, size-exclusion chromatography (SEC), and Ellman’s assay for thiol concentration [28]. For a detailed description of the polymer synthesis, we refer to a previous publication [25]. The monomers required for the synthesis of the polymers were inexpensive and could be prepared in a single synthesis step in large quantities. The composition of the polymers is shown in Table 1.

The bifunctional, disulfide-containing monomer BMAC leads to an in situ formation of the gel during polymerization. This leads to a very uncontrolled polymerization, which explains the deviation of monomer ratios from NMR spectroscopy compared to the theoretical composition. Nevertheless, a lower concentration of thiols compared to the theoretical concentration was sufficient to form gels. To study the influence of peptides, first an unfunctionalized polymer was prepared with an increased BMAC concentration of 7.5 mol%, which was then partially reacted with the maleimide-containing peptide sequence MIC-6AHX-YGRGDS via a Michael reaction. In this process, 3.5 mol% of the initial 7.5 mol% BMAC should be functionalized; however, we found a degree of functionalization of 4.8 mol% t via ^1^H-NMR spectroscopy after the reaction.

### 2.2. Preparation of the Hydrogels

**Scheme 1:**
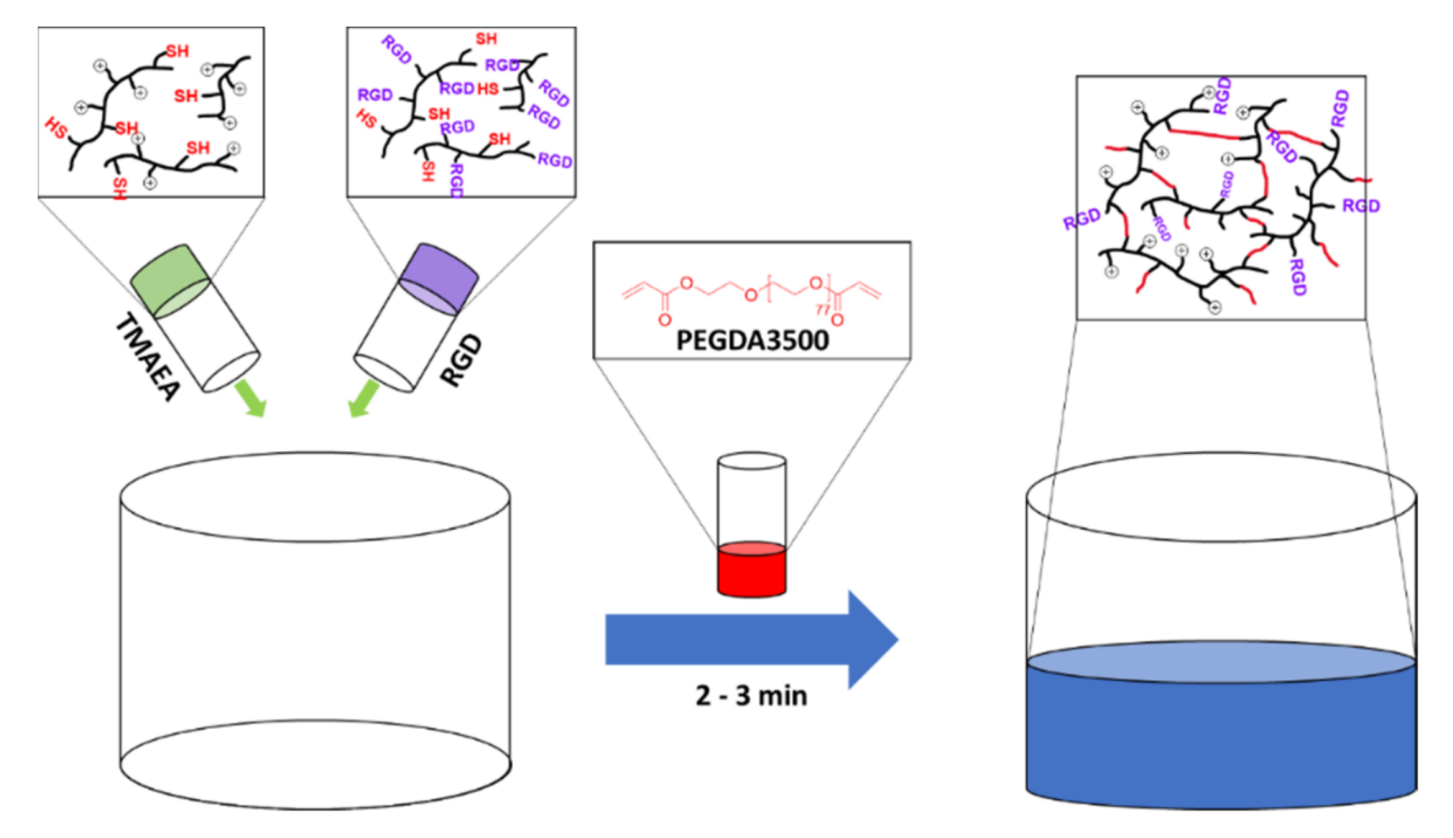
Schematic overview of the preparation of the mixed gel G4 from a separate cationic prepolymer **P2** and an RGD-containing prepolymer **P3** crosslinked with PEGDA3500.

The hydrogels were prepared via a thiol-ene reaction of the thiol-containing prepolymers with the acrylate groups of PEGDA3500. The deprotonated thiols of the prepolymers react with the double bond of the acrylate group of PEGDA3500 in a nucleophilic attack via a Michael addition pathway resulting in a new C–S bond to form a crosslink. Three different types of gels were prepared: gels without other functions (G1), cationic gels (G2, G4–G8), and RGD-peptide functionalized gels (G3, G4). All hydrogels were prepared by combining separate prepolymer solutions with the PEGDA3500 solution in defined ratios (Scheme 1). The gelation time for all hydrogels were between 2 and 3 min independent of the concentration of cationic groups or RGD peptide. For a more detailed study of the gelation time, we refer to a previous study [25].

A total of eight different gels were used, and the corresponding data of the gels can be found in Table 2. Since the mechanical properties of the gels also have an impact on the behavior of the cells, it is necessary to adjust the stiffness of the gels to the corresponding cell line. While neural cells prefer rather soft tissue, cells from the bones need a rather hard matrix [29]. The storage modulus could be adapted to the cells under investigation via the gel composition, the degree of crosslinking of the thiols, and the mass fraction used for gelation [25]. 

Interestingly, all gels produced showed similar swelling behavior in phosphate buffered saline (PBS). Even though a cationic group with a permanent cationic charge, which was very hydrophilic, was used, G2 with the highest concentration of 0.91 µmol/mg did not show a significant increase in the swelling behavior. One reason for this observation could be the usage of PBS during the preparation of hydrogels and for swelling of the gels. Phosphates are known for their high affinity towards quaternary ammonium groups and ammonium ions are often used in the literature for the removal of phosphates from solutions [30,31]. Since phosphates are abundant in PBS buffer, every ammonium group is surrounded with phosphate ions and therefore the hydrophilic cationic charge of the ammonium group was shielded resulting in a similar swelling of all gels bearing cationic groups despite their different concentrations. Swelling of cationic gels in water led to a concentration-dependent swelling behavior with the highest swelling factor for the highest cationic concentration and the lowest swelling factor for the lowest concentration, respectively, supporting our previous thesis. As shown in Table 2, the Young’s modulus *E* averaged at 9.1 kPa with the lowest E-modulus of 7.7 kPa for G1 and the highest E-modulus of 11.1 kPa for G6 (the gels G3 and G4 containing the peptide RGD were evaluated separately due to their lower concentration of 10 wt% during preparation). All hydrogels were stable and showed no change of mechanical properties over the course of the experiments. There were no clear trends visible for the cationic gels, indicating that the latter effects on cell cultivation are independent of the swelling behavior and mechanical properties of the gels and were indeed an effect of the concentration of cationic groups. The ζ-potential of free polymers showed the highest ζ-potential of +42.1 mV for polymer P2 possessing the highest concentration of cationic groups of 0.91 µmol/mg. If this polymer was mixed with P1, i.e., a polymer without a cationic group, the ζ-potential would decrease to +20.0 mV for the lowest concentration of 0.06 µmol/mg. This data showed the decrease of ζ-potential upon lowering the concentration of cationic groups. The slightly negative ζ-potential of −3.9 mV for the unfunctional polymer can be explained by the deprotonated thiols present at pH = 7.4. A gel with these polymers is expected to have a ζ-potential close to zero, since most of the thiols were used to crosslink the gel. The cationic gels, on the other hand, have a permanent cationic charge and therefore always exhibit a positive ζ-potential.

In addition to the cationic gels, two more gels (G3 and G4) containing the RGD sequence were fabricated and the influence on the adhesion of U-251-MG cells to these hydrogels was evaluated. These gels were an exception compared to the cationic gels because they were formed in a 10 wt% solution compared to the 18 wt% solution used for other gels. A change in the mass fraction for these gels highly impacted the Young’s modulus as can be seen with the 4–5 times lower E-modulus of around 2.1 kPa compared to the 18 wt% gels. This was due to lower crosslinking of these gels leading to lower E-modulus and therefore a higher swelling. In addition, the total amount of cationic groups inside the gel was lower compared to 18 wt% gels. The ζ-potential of the cationic peptide polymer showed a positive ζ-potential of +36.6 mV which is in accordance with the other cationic gels. The pure peptide gel G3 exhibited a highly negative ζ-potential of −15.4 mV due to the presence of multiple hydroxyl groups and carboxylic acids in the peptide. Nevertheless, these two gels could be used to compare the effects of peptides and the combination of peptide and cationic groups on cell cultivation.

**Figure 2:**
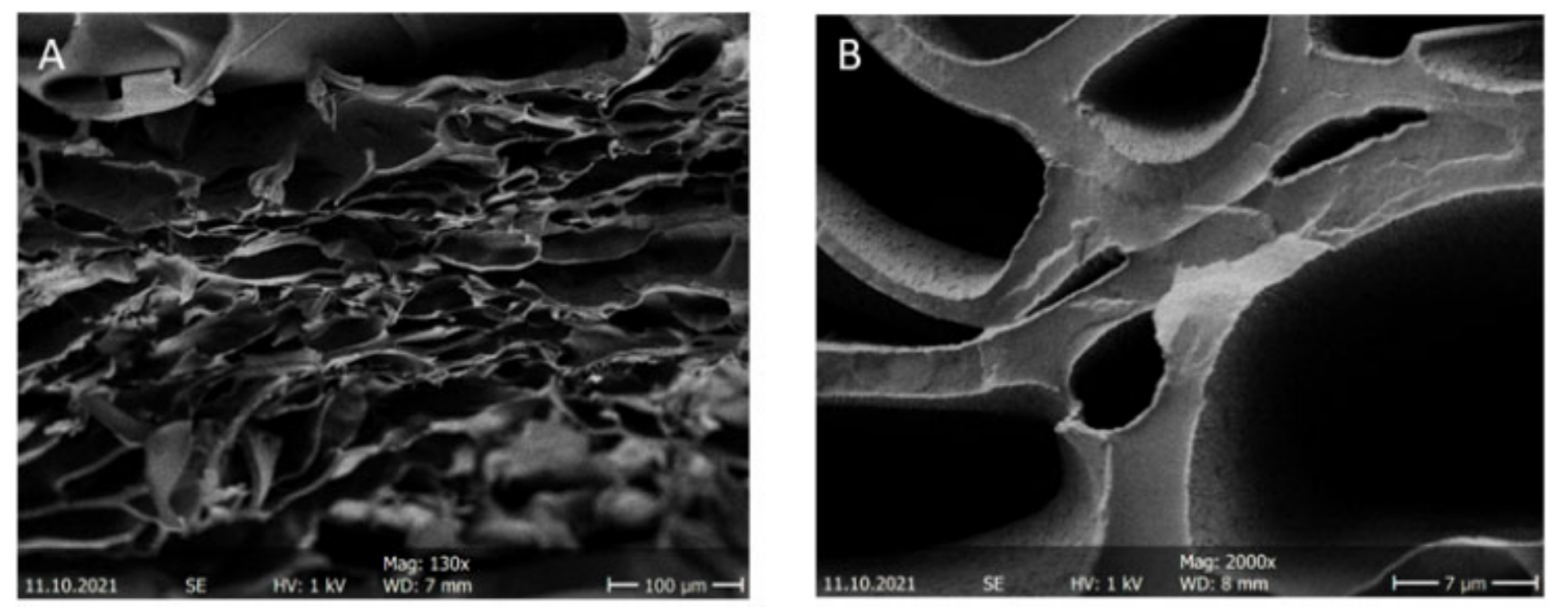
SEM images of the section of the 18 wt% cationic hydrogel G2 at (**A**) a 100 µm scale and (**B**) a 7 µm scale.

Lastly, scanning electron microscope (SEM) images of the gel with a cationic concentration of 0.91 µmol/mg were taken. These gels were initially completely swollen gels, which were dried via freeze-drying (Figure 2A,B). A completely porous internal structure of the gel can be seen, with pores of different sizes. It should be noted that these images are taken of dried gel samples and most likely do not resemble the polymer structure of the gel in a fully swollen state.

### 2.3. The Interplay of Cationic Charge and Bioactive Motif on Adhesion of U-251-MG Cells

**Figure 3:**
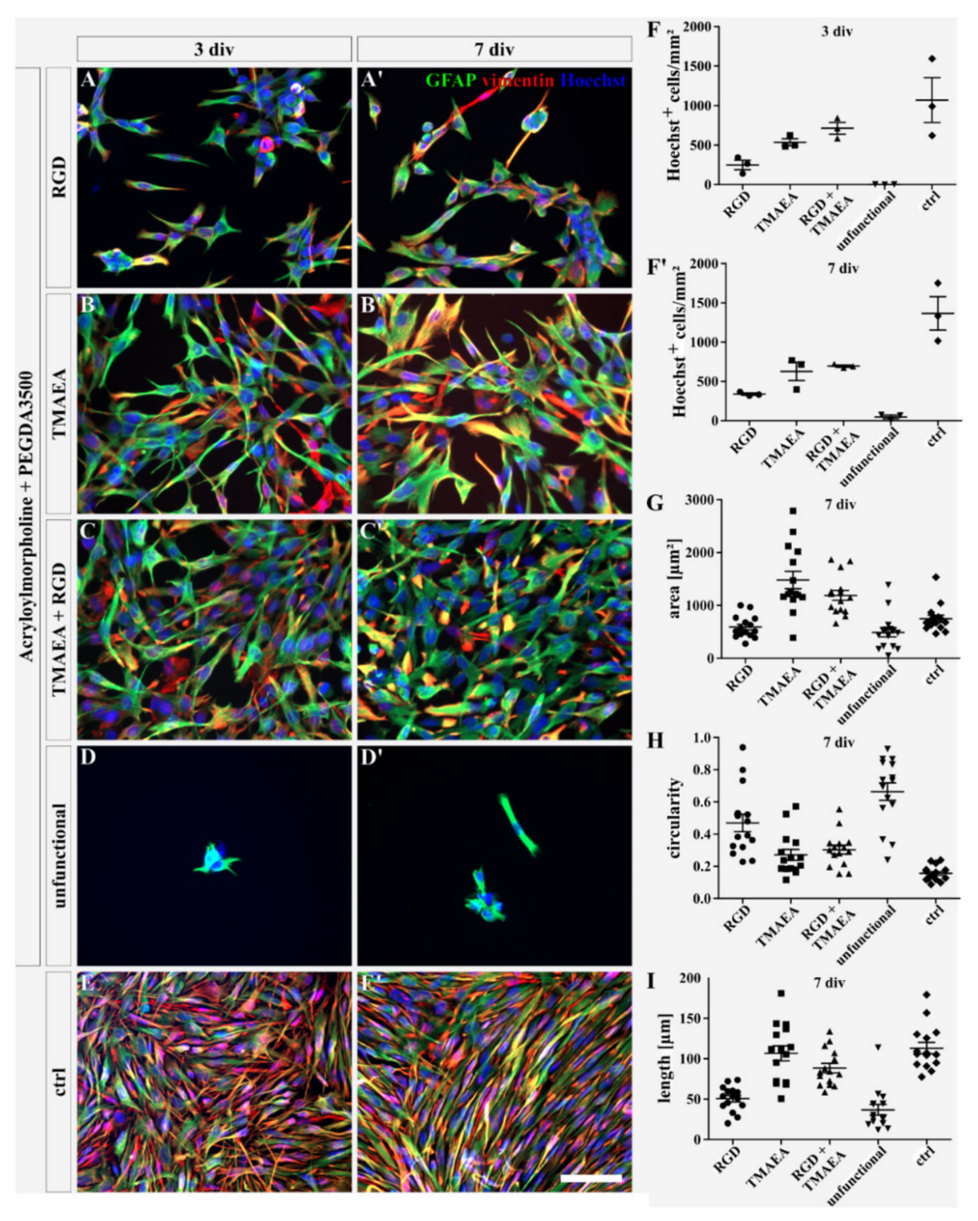
Fluorescence images of U-251-MG cells cultured on hydrogels G1–G4 containing the RGD peptide sequence (G3, **A**,**A′**), the cationic monomer TMAEA (G2, **B**,**B′**), a combination of peptide and cationic group (G4, **C**,**C′**), or no further functionalization (G1, **D**,**D′**) after 3 and 7 days. The control (**E**,**E′**) displays U-251-MG cells cultured on uncoated cell culture dishes. The proteins GFAP (green) and vimentin (red) are intermediate filaments and reflect the morphology of the cells. Hoechst (blue) marks the cell nuclei. Enumeration of Hoechst-positive cells on the gels G1–G4 after 3 (**F**) and 7 (**F′**) days of cultivation, respectively, determines the total number of cells on the hydrogel within 1 mm^2^. The cell spreading area (**G**), circularity (**H**), and length (**I**) were calculated after 7 div whereat 15 cells were measured in each condition. (scale bar: 100 µm; mean ± SEM; N = 1, n = 3 [Hoechst -positive cells]; N = 1, n = 15 [area, circularity, length]).

For neural stem cells (NSCs), Saha et al. previously demonstrated that a lateral concentration greater than 5.3 pmol/cm^2^ of an RGD peptide is necessary to support adhesion and differentiation on interpenetrated polymer networks [32]. Sallouh and Jarocki et al. designed a hydrogel system containing both a cationic motif and the RGD peptide well below the reported concentration by Saha et al. The combination of both motifs showed a synergistic effect on NSC adhesion, where the number of adherent cells could be increased even with low concentrations of the RGD peptide [16]. This synergistic effect is described as a two-step process. Adhesion of cells normally occurs in the first hours after the start of cultivation and the cationic charge promotes the initial contact of the cells with the surface of the gel. In the second step, the connection of the RGD peptide with cell receptors such as integrins is built. As an introducing pilot study, we repeated these experiments to see whether the same synergistic effect could be seen in this novel synthetic hydrogel system for the U-251-MG cell line as well. U-251-MG cells, a robust and well-established human glioblastoma cell line, were used to evaluate the gel system and the effects of different motifs on cell cultivation. For this purpose, the pure peptide gel G3 with a total concentration of 0.26 µmol/mg peptide and a mixed gel G4, in which P3 and P2 were mixed in a ratio of 1:1, resulting in a hydrogel with a concentration of 0.13 µmol/mg peptide and 0.63 µmol/mg cationic group, were used. Figure 3 shows the fluorescence staining against glial fibrillary acidic protein (GFAP; green) and vimentin (red) to represent the morphology of the U-251-MG cells on G1–G4. The pure RGD gel (G3; Figure 3A,A′) showed the adherence of single cells with a plane morphology and small cell bodies after 3 and 7 days in vitro (div) (Figure 3G–I, area: 590.51 ± 54.52 µm^2^; circularity: 0.47 ± 0.05; length: 50.51 ± 4.02 µm). Additionally, swimming sphere-like structures were found above the hydrogel, indicating weak adhesion capability. A higher cell number was detected on the cationic hydrogel with a concentration of 1.29 µmol/mg TMAEA (G2; Figure 3B,B′; Figure 3F,F′; G2 3 div: 535 ± 44.7; G2 7 div: 627.7 ± 115.8; G3 3 div: 249.3 ± 59.7; G3 7 div: 339 ± 14.7). Additionally, the cell spreading area was increased on this hydrogel including a more stretched morphology and less circularity compared with the RGD gel (Figure 3G–I; area: 1479.01 ± 162.16 µm^2^; circularity: 0.27 ± 0.03; length: 106.78 ± 9.34 µm). The combined gel with RGD peptide and the cationic motif TMAEA (G4; Figure 3C,C′) showed a synergistic effect comparable to the one reported by Sallouh and Jarocki [16] which resulted in the appearance of a cell layer comparable to the control (Figure 3E,E′). The cells presented slightly smaller cell bodies with a less elongated morphology than the cells on the pure cationic hydrogel (Figure 3G–I; area: 1189.47 ± 96.14 µm^2^; circulation: 0.3 ± 0.03; length: 88.39 ± 5.83 µm). The Hoechst-positive nuclei exhibited a slight increase in the number of cells (Figure 3F,F′; 3 div: 713.7 ± 74.4; 7 div: 695.3 ± 14.3) compared to the pure cationic gel with a concentration of 1.29 µmol/mg. On the unfunctional hydrogel (G1), cells adhered only rarely (Figure 3D,D′; 3 div: 2 ± 0; 7 div: 71.5 ± 4.5) and the morphology of the cells was smaller and less stretched (Figure 3G–I; area: 491.75 ± 89.12 µm^2^; circularity: 0.66 ± 0.05; length: 36.8 ± 6.56 µm). The cells on the control, which was an uncoated cell culture dish, appeared in an elongated and stretched morphology with an increased cell body length but a smaller cell spreading area than on the cationic and the mixed hydrogel (Figure 3G–I; area: 749.59 ± 67.39 µm^2^; circularity: 0.16 ± 0.01; length: 113.12 ± 7.12 µm). Nevertheless, these cultures indicated the highest cell number (Figure 3F,F′; 3 div: 1068.67 ± 283.55; 7 div: 1366 ± 212.57). This pilot study confirms the capabilities of this novel synthetic hydrogel system to support adhesion of U-251-MG cells.

### 2.4. The Concentration Dependant Effect of the Cationic Motif TMAEA on Cell Adhesion of U-251-MG Cells

**Figure 4:**
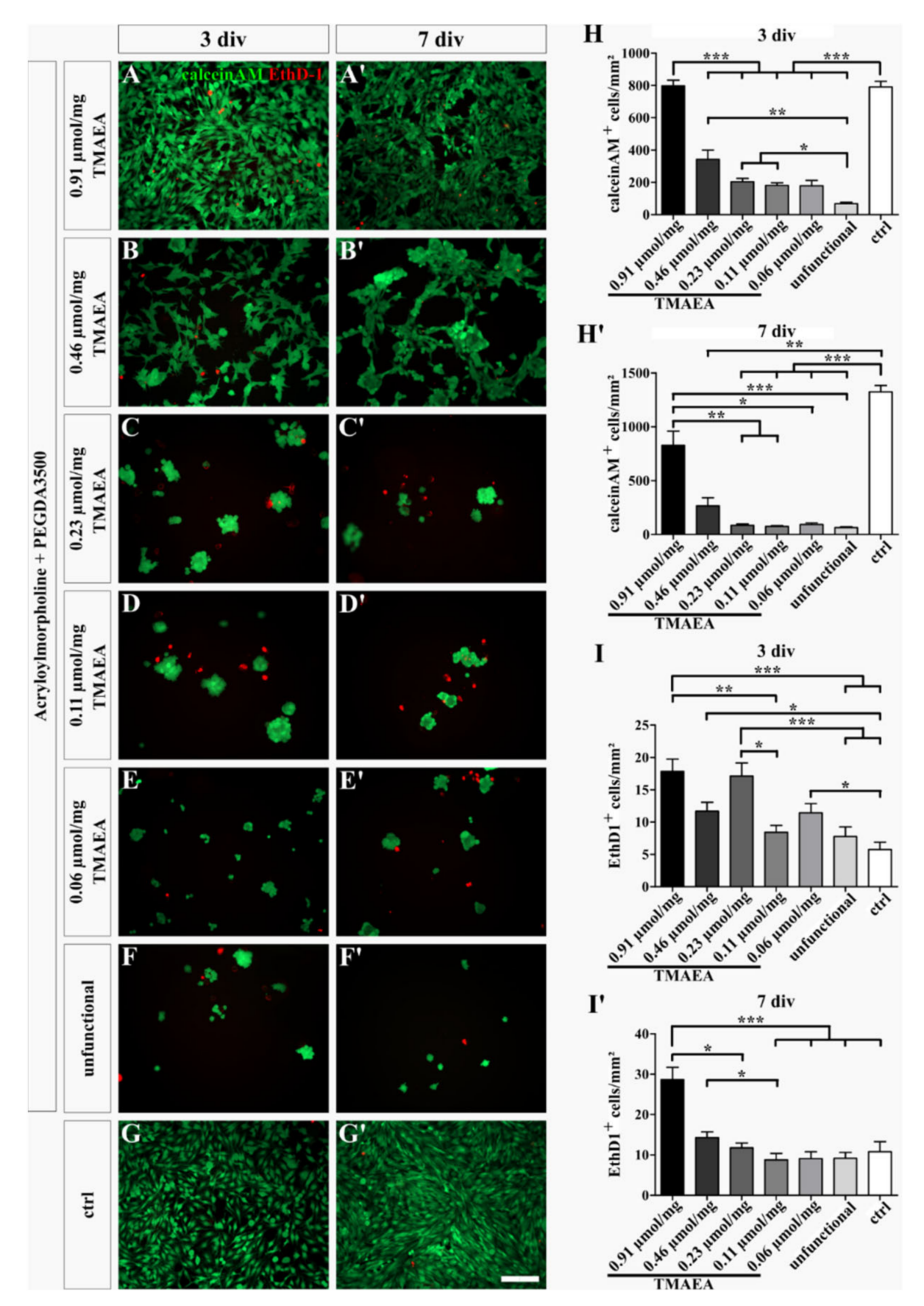
Live/dead staining of U-251-MG cells on hydrogels with different concentrations of the cationic motif TMAEA (G8 = 0.06 μmol/mg; G7 = 0.11 μmol/mg; G6 = 0.23 μmol/mg; G5 = 0.46 μmol/mg; G2 = 0.91 μmol/mg). The fluorescence images show living (calcein-AM, green) and dead (EthD-1, red) cells after 3 days (**A**–**G**) and 7 days (**A′**–**G′**) of cultivation. Uncoated cell culture dishes were used as a control (**G**,**G′**). The graphs indicate the number of living (**H**,**H′**) and dead (**I**,**I′**) cells after 3 and 7 days of cultivation within 1 mm^2^. (scale bar: 200 µm; mean ± SEM; N = 3, n = 24; Kruskal–Wallis test with *post hoc* Dunn’s test; * *p* ≤ 0.05, ** *p* ≤ 0.01, *** *p* ≤ 0.001).

The effect of different concentrations of the cationic motif TMAEA on the survival of U-251-MG cells was evaluated. We described a total concentration of cationic groups per mg of hydrogel to compare the effect of cationic groups on adhesion and survival (see Table 2). A total of five different concentrations were tested between 0.91 µmol/mg (G2) as the highest concentration and 0.06 µmol/mg (G8) as the lowest concentration. In the first experiment, we investigated the viability of U-251-MG cells on these hydrogels. The survival of the cells was analyzed via a live/dead staining using the dye calcein-AM for living cells (green) and ethidium homodimer-1 (EthD-1; red) to mark dead cells (Figure 4). The quantification exhibited that the total number of adherent cells was dependent on the concentration of the cationic group. After 3 div, the number of living cells on the hydrogel with a 0.91 µmol/mg TMAEA concentration (797 ± 36.19) was as high as on the control (790.3 ± 35.48) and both were significantly higher than the number of living cells on the other hydrogels (0.46 µmol/mg: 342.6 ± 56.97; 0.23 µmol/mg: 204.6 ± 20.13; 0.11 µmol/mg: 181.4 ± 15.71; 0.06 µmol/mg: 178.7 ± 33.09; unfunctional: 67.5 ± 9.29). Furthermore, the hydrogels with a high or medium TMAEA concentration, namely 0.46 µmol/mg, 0.23 µmol/mg, and 0.11 µmol/mg, harbored significantly more living cells than the unfunctional hydrogel (Figure 4H). After 7 div, the number of living cells on the control (1325 ± 59.2) increased over time, whereas the number of living cells on the hydrogels decreased during this period. However, the control displayed the most living cells and a significantly increased cell number compared to the hydrogels with a TMAEA concentration of 0.46 µmol/mg (265.8 ± 76.38), 0.23 µmol/mg (86 ± 12.65), 0.11 µmol/mg (74.29 ± 9.433), 0.06 µmol/mg (92.54 ± 14.79), and the unfunctional (63.88 ± 8.487) hydrogel. The hydrogel with the highest TMAEA concentration contained significantly more living cells (828.3 ± 130.7) compared to the hydrogels with a medium or low TMAEA concentration, namely 0.46 µmol/mg, 0.23 µmol/mg, 0.11 µmol/mg, 0.06 µmol/mg TMAEA, and the unfunctional hydrogel (Figure 4H′).

Additionally, the number of dead cells was quantified (Figure 4I,I′). By comparing the different conditions, it was noticed that the lowest number of dead cells was found in the control (3 div: 5.75 ±1.139; 7 div: 10.79 ± 2.538). Surprisingly, the hydrogel with the highest TMAEA concentration (3 div: 17.83 ± 1.916; 7 div: 28.67 ± 3.049) displayed a significantly increased death rate compared to the control after 3 and 7 days and other hydrogels, namely the 0.11 µmol/mg TMAEA (8.417 ± 1.065) and unfunctional hydrogel (7.792 ± 1.467) after 3 div and the 0.23 µmol/mg (11.75 ± 1.249), 0.11 µmol/mg (8.792 ± 1.583), 0.06 µmol/mg TMAEA (9.125 ± 1.659), and the unfunctional hydrogel (9.167 ± 1.455) after 7 div. Additionally, the hydrogel with a concentration of 0.46 µmol/mg TMAEA (3 div: 11.67 ± 1.416; 7 div: 14.33 ± 1.387) contained significantly more dead cells after 3 div compared to the control (5.792 ± 1.467) and after 7 div compared to the 0.11 µmol/mg TMAEA hydrogel (8.792 ± 1.583). After 3 div, the number of dead cells on the hydrogel at 0.23 µmol/mg TMAEA (17.13 ± 2.011) was almost as high as on the hydrogel with the highest concentration of 0.91 µmol/mg TMAEA (17.83 ± 1.916) and thus significantly higher than on the hydrogel at 0.11 µmol/mg TMAEA (8.417 ± 1.065), the unfunctional hydrogel (7.792 ± 1.467), and the control (5.75 ± 1.139). Furthermore, the hydrogel at 0.06 µmol/mg TMAEA (11.42 ± 1.456) showed significantly more dead cells than the control (5.75 ± 1.139) after 3 div. The increase of dead cells adherent to the positively charged hydrogels is most likely due to the unspecific interaction of the cationic charge of TMAEA with the negatively charged cell residuals. Therefore, not only the adhesion of living cells but dead cells as well was increased.

**Figure 5:**
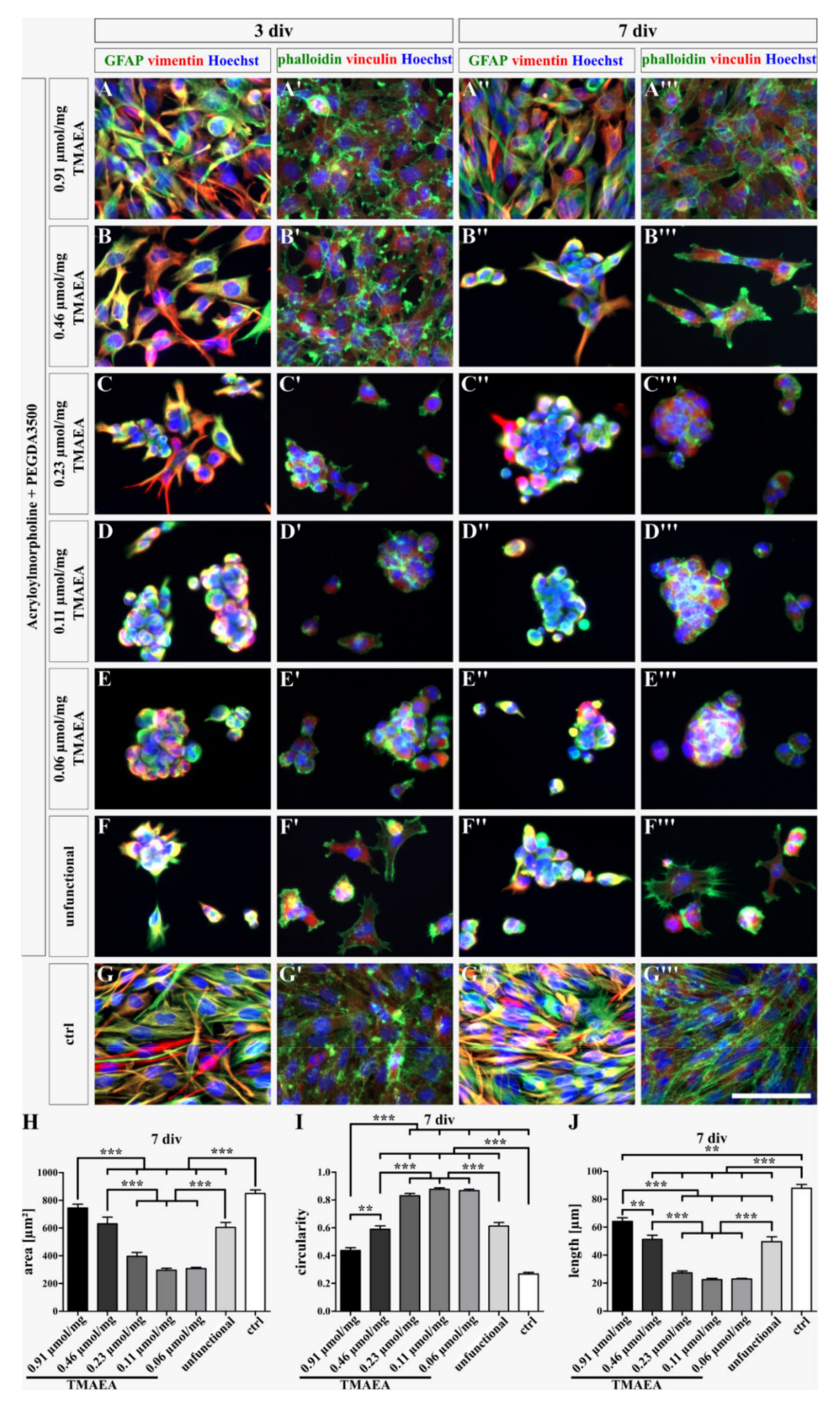
Fluorescence images of U-251-MG cells cultured on hydrogels with different cationic concentrations (G8 = 0.06 μmol/mg; G7 = 0.11 μmol/mg; G6 = 0.23 μmol/mg; G5 = 0.46 μmol/mg; G2 = 0.91 μmol/mg, (**A**–**G**,**A′**–**G′**) after 3 days of culturing, (**A″**–**G″**,**A‴**–**G‴**) after 7 days of culturing). The control displays U-251-MG cells on uncoated cell culture dishes. The proteins GFAP (green), vimentin (red), and vinculin (red) are intermediate filaments. Phalloidin (green) labels F-actin is a microfilament. Hoechst (blue) is a marker for cell nuclei. All stained proteins are part of the cytoskeleton and show the morphology of the cells. The cell spreading area (**H**), circularity (**I**), and length (**J**) were calculated after 7 div whereat 120 cells were measured in each condition. (scale bar: 100 µm; mean ± SEM; N = 3, n = 120; Kruskal–Wallis test with post hoc Dunn’s test; * *p* ≤ 0.05, ** *p* ≤ 0.01, *** *p* ≤ 0.001.

Furthermore, we evaluated the morphology of adherent U-251-MG cells on the hydrogel. During adhesion of the U-251-MG cells to surfaces, the cells form plane elongated or stretched morphologies [33]. Figure 5 shows the staining of the cytoskeleton, namely the intermediate filaments GFAP (green), vimentin (red), and vinculin (red), as well as F-actin, which is marked by phalloidin (green). It was noticed that on the hydrogels with a concentration of 0.06 µmol/mg, 0.11 µmol/mg, and 0.23 µmol/mg TMAEA, the cells tended to form clusters and showed a round morphology after 3 and 7 div (Figure 5C–C‴,D–D‴,E–E‴,H–J; 0.06 µmol/mg TMAEA: area: 308.14 ± 9.11 µm^2^; circularity: 0.87 ± 0.01; length: 22.93 ± 0.57 µm; 0.11 µmol/mg TMAEA: area: 296.53 µm^2^; circularity: 0.88 ± 0.01; length: 22.54 ± 0.86 µm; 0.23 µmol/mg TMAEA: area: 395.99 µm^2^; circularity: 0.83 ± 0.02; length: 27.25 ± 1.43 µm). However, sporadic single cells were found after 3 div. At a concentration of 0.46 µmol/mg TMAEA, most cells adhered separately on the hydrogel and showed a stretched morphology after 3 div, but after 7 div, cell clusters were found (Figure 5B–B‴). However, separated cells exhibited a significantly increased cell spreading area and length as well as a decreased circularity compared to the hydrogels at 0.06 µmol/mg, 0.11 µmol/mg, and 0.23 µmol/mg TMAEA (Figure 5H–J; area: 630.71 ± 47.96 µm^2^; circularity: 0.59 ± 0.02; length: 51.19 ± 3.11 µm). A similar morphology of the U-251-MG cells was found in the cultures on the unfunctional hydrogel after 7 div (Figure 5F–F‴,H–J; area: 604.85 ± 35.31 µm^2^; circularity: 0.61 ± 0.03; length: 49.6 ± 3.59 µm). Remarkably, the gel surface was completely covered by cells with a stretched morphology at a concentration of 0.91 µmol/mg TMAEA with a comparable appearance of the cells on the surface of the control which was an uncoated cell culture dish (Figure 5A–A‴,G–G‴,H–J; 0.91 µmol/mg TMAEA: area: 744.34 ± 27.53 µm^2^; circularity: 0.44 ± 0.02; length: 64.12 ± 2.6 µm; control: area: 849.84 ± 24.65 µm^2^; circularity: 0.27 ± 0.01; length: 87.9 ± 2.67 µm). In both cultures, the cell spreading area and length was significantly increased and the circularity was significantly decreased compared to the other hydrogels. Accordingly, we hypothesized a minimum cationic concentration of 0.46 µmol/mg was necessary to promote adhesion of the U-251-MG cells on the novel synthetic hydrogels. Gels with a lower concentration of cationic groups showed a low number of adherent cells which formed clusters.

### 2.5. Comparison of Dialyzed and Undialyzed Gels

**Figure 6:**
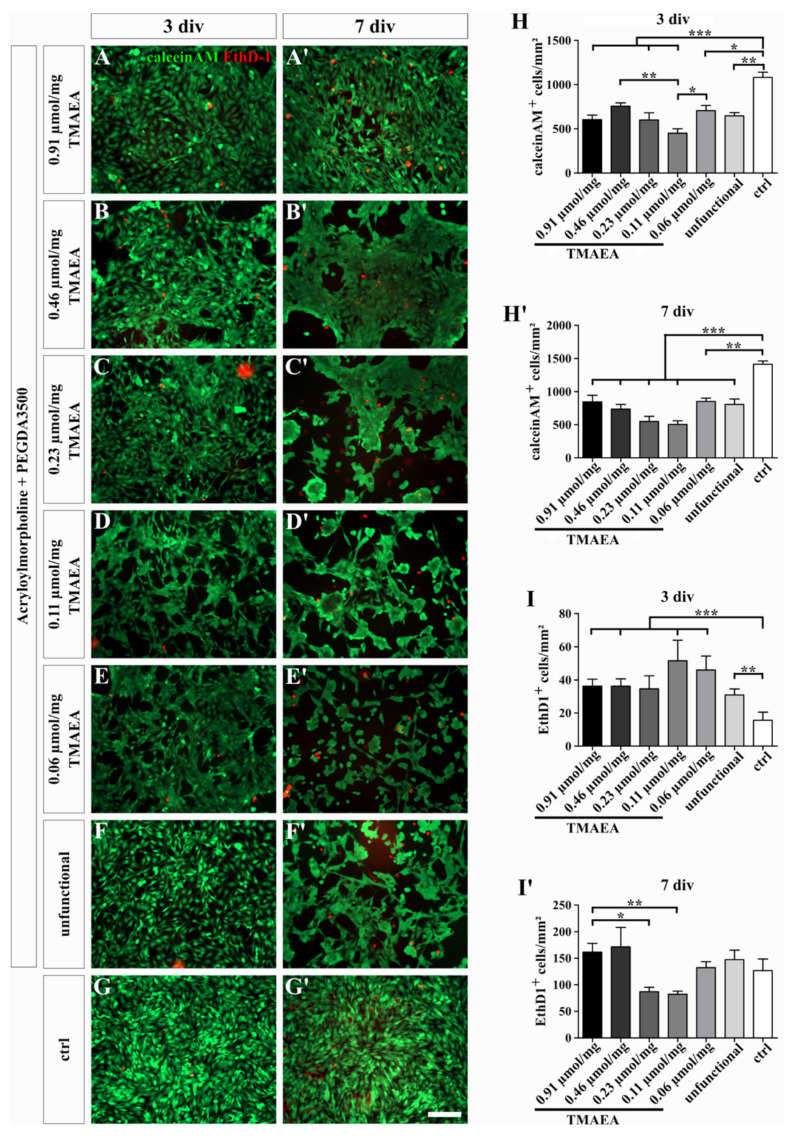
Live/dead staining of U-251-MG cells on dialyzed hydrogels with different concentrations of the cationic motif TMAEA (G8 = 0.06 μmol/mg; G7 = 0.11 μmol/mg; G6 = 0.23 μmol/mg; G5 = 0.46 μmol/mg; G2 = 0.91 μmol/mg). The fluorescence images show living (calein-AM, green) and dead (EthD-1, red) cells after 3 days (**A**–**G**) and 7 days (**A′**–**G′**) of cultivation. Uncoated cell culture dishes were used as a control (**G**,**G′**). The graphs indicate the number of living (**H**,**H′**) and dead (**I**,**I′**) cells after 3 and 7 days of cultivation within 1 mm^2^. (scale bar: 200 µm; mean ± SEM; N = 3, n = 24; Kruskal–Wallis test with *post hoc* Dunn’s test; * *p* ≤ 0.05, ** *p* ≤ 0.01, *** *p* ≤ 0.001).

**Figure 7:**
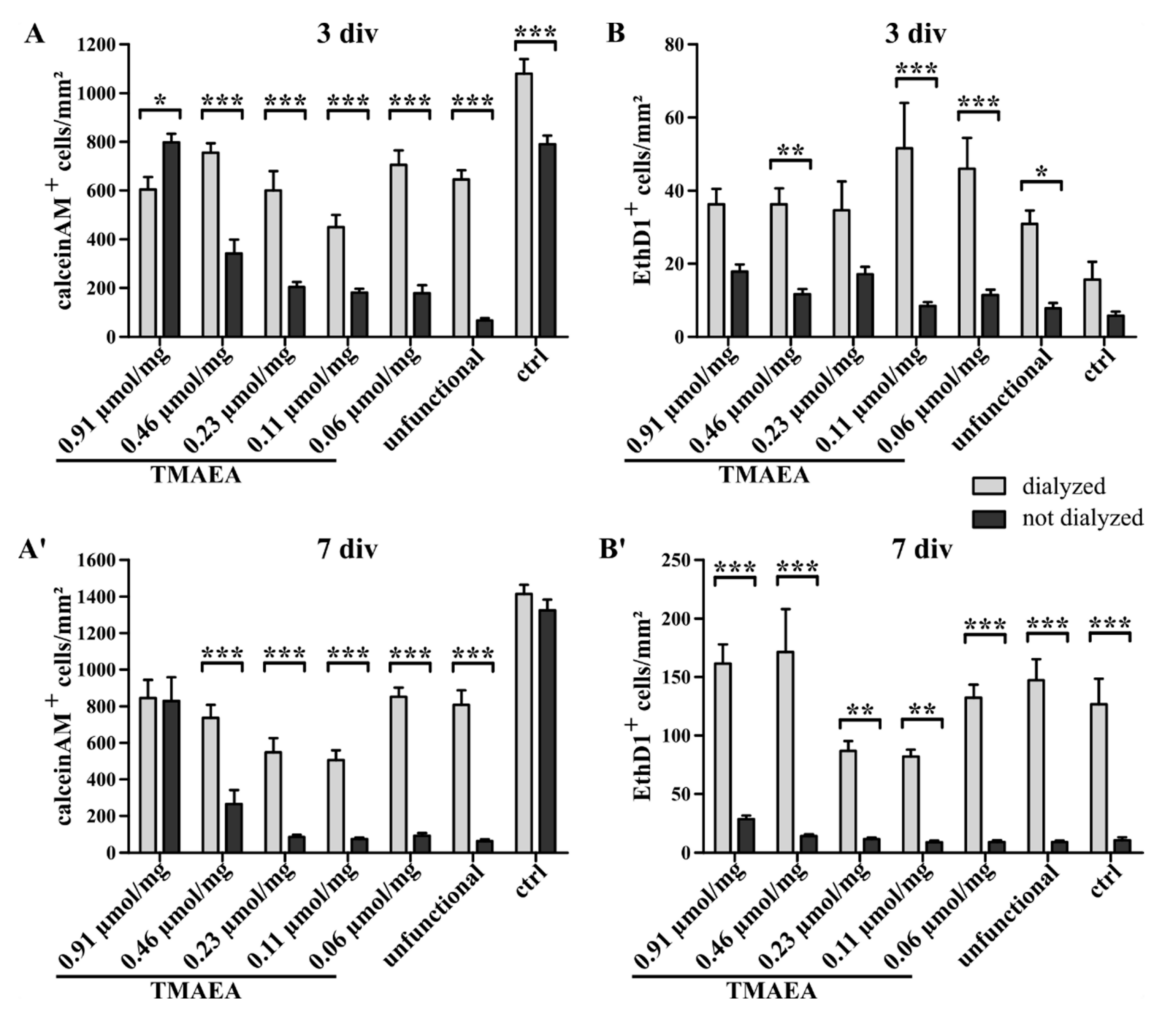
Comparison of the quantification of the live/dead staining of U-251-MG cells on dialyzed and undialyzed hydrogels containing different concentrations of the cationic motif TMAEA (G8 = 0.06 μmol/mg; G7 = 0.11 μmol/mg; G6 = 0.23 μmol/mg; G5 = 0.46 μmol/mg; G2 = 0.91 μmol/mg) after 3 days of cell culture (**A**,**B**) and 7 days of cell culture (**A′**,**B′**), (mean ± SEM; N = 3, n = 24; two-way ANOVA with post hoc Bonferroni test; * *p* ≤ 0.05, ** *p* ≤ 0.01, *** *p* ≤ 0.001).

Many types of cells are prone to small impurities in their environment leading to a reduced capability of cells to survive during cultivation. To increase the survival rate of the U-251-MG cells and to promote the normal outstretched morphology, the hydrogels were dialyzed in water for several days to remove any residues such as free polymer chains or minimal amounts of solvents left from the synthesis which might have a negative influence on the cultivation of cells. The portion of living and dead cells on the dialyzed hydrogels was evaluated with a live/dead staining using the dye calcein-AM (green) as a marker for living cells and EthD-1 (red) as a stain for dead cells (Figure 6). The quantification revealed that the control (1080 ± 60.19) harbored significantly more living cells than the hydrogels (0.91 µmol/mg: 605 ± 50.38; 0.23 µmol/mg: 601.1 ± 78.98; 0.11 µmol/mg: 450.5 ± 49.84; 0.06 µmol/mg: 705.8 ± 59.81; unfunctional: 645.9 ± 37.27) after 3 div, except for the hydrogel at 0.46 µmol/mg TMAEA (755.9 ± 39.07). This hydrogel contained significantly more living cells than the lower charged hydrogel at 0.11 µmol/mg TMAEA which also included significantly less cells than the low charged hydrogel at 0.06 µmol/mg TMAEA (Figure 6H). After 7 div, the number of living cells in the control (1413 ± 50.99) was again significantly higher compared to the hydrogels (Figure 6H′; 0.91 µmol/mg: 845.5 ± 99.68; 0.46 µmol/mg: 736.4 ± 71.85; 0.23 µmol/mg: 548.6 ± 77.29; 0.11 µmol/mg: 506 ± 53.98; 0.06 µmol/mg: 850.7 ± 52; unfunctional: 807.5 ± 79.72). The quantification of the portion of dead cells after 3 div revealed that significantly less EthD-1-positive cells were found in the control (15.67 ± 4.913) compared to most hydrogels, namely at 0.91 µmol/mg (36.33 ± 4.175), 0.46 µmol/mg (36.25 ± 4.377), 0.11 µmol/mg (51.58 ± 12.39), 0.06 µmol/mg TMAEA (46.04 ± 8.385), and the uncharged hydrogel (30.88 ± 3.681) (Figure 6I). After 7 div, the hydrogel with the highest concentration of TMAEA (161.5 ± 16.6) contained significantly more dead cells compared to the hydrogels with a concentration of 0.23 µmol/mg (86.92 ± 8.422) and 0.11 µmol/mg TMAEA (82.08 ± 5.972) (Figure 6I′). While the previous experiment with undialyzed gels indicated that the adhesion is dependent on the concentration of cationic groups, this experiment showed the importance of working with pure materials and that small amounts of unbound polymers or solvents highly reduce the viability of cells. Except for G2 with the highest concentration of 0.91 µmol/mg, every dialyzed gel displayed a higher number of total cells adherent to the surface. In our studies, it seemed that the dialyzed hydrogels with low or medium TMAEA concentration showed good viability of cells comparable to G2. A comparison of the total number of cells (Figure 7) showed a strong effect of the dialysis, especially for lower concentrations. Interestingly, the number of cells for the highest cationic concentration does not increase when using dialysis. This indicates a superior role of the cationic group compared to using dialysis when the cationic concentration is high enough, which results in no further increase in the total number of cells. We hypothesized that the cells need a higher degree of support in the form of adhesion motifs such as the TMAEA molecule we used in our hydrogels when cultured on undialyzed hydrogels. These hydrogels possessed disturbing factors such as unbound polymers or solvents which have a detrimental effect on cell adhesion and thus partially neutralize the supportive effect of TMAEA. When these obstacles were overcome through the dialysis of the hydrogels, a lower concentration of TMAEA was sufficient for the adhesion of the U-251-MG cells.

**Figure 8:**
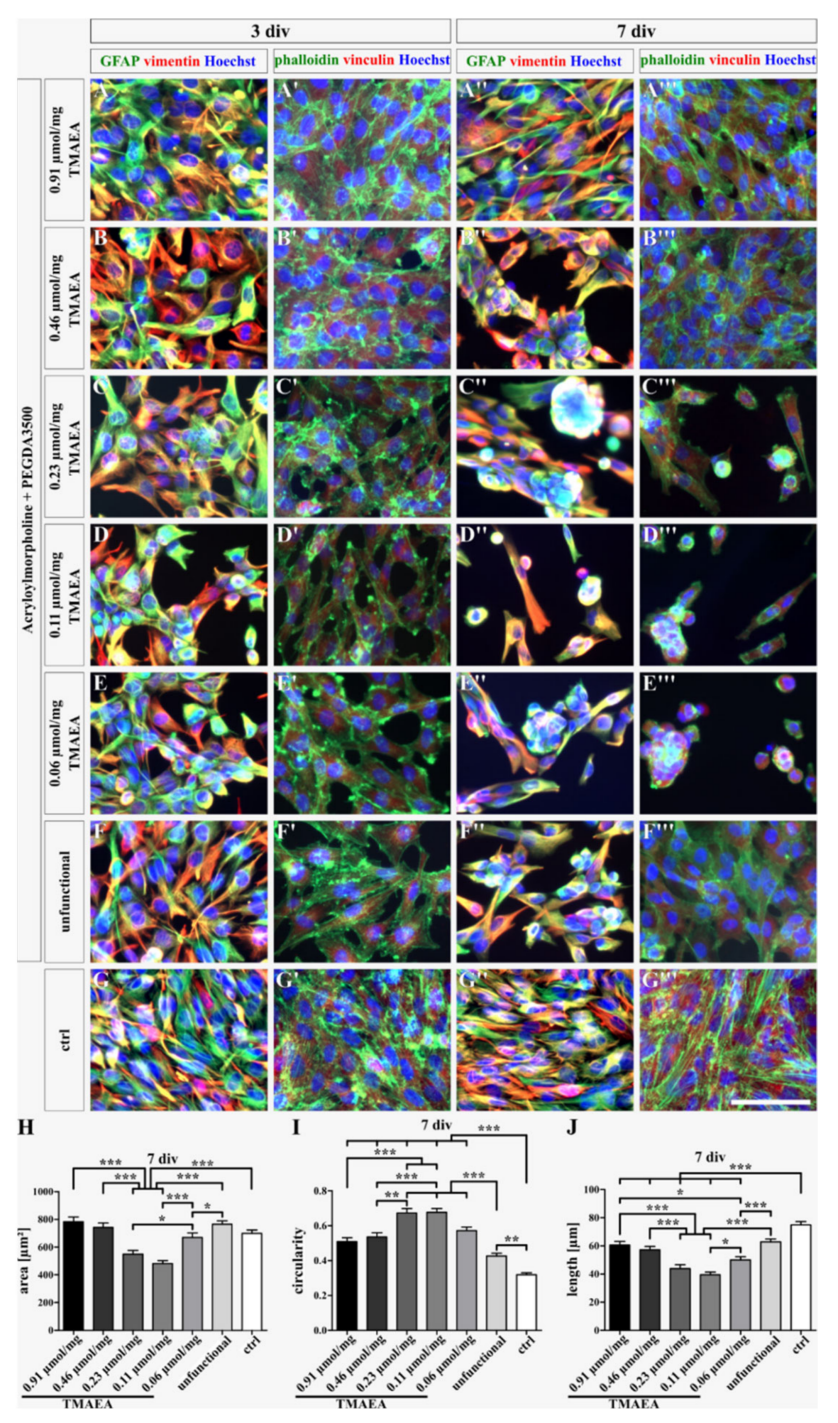
Fluorescence images of U-251-MG cells cultured on dialyzed hydrogels with different cationic concentrations (G8 = 0.06 μmol/mg; G7 = 0.11 μmol/mg; G6 = 0.23 μmol/mg; G5 = 0.46 μmol/mg; G2 = 0.91 μmol/mg, after 3 days of cell culturing (**A**–**G**,**A′**–**G′**) and 7 days of cell culturing (**A″**–**G″**,**A‴**–**G‴**). The control displays U-251-MG cells on uncoated cell culture dishes. The proteins GFAP (green), vimentin (red), and vinculin (red) are intermediate filaments. Phalloidin (green) is a marker for F-actin, which is a microfilament. Hoechst (blue) is a marker for cell nuclei. All stained proteins are part of the cytoskeleton and show the morphology of the cells. The cell spreading area (**H**), circularity (**I**), and length (**J**) were calculated after 7 div whereat 120 cells were measured in each condition. (scale bar: 100 µm; mean ± SEM; N = 3, n = 120; Kruskal–Wallis test with post hoc Dunn’s test; * *p* ≤ 0.05, ** *p* ≤ 0.01, *** *p* ≤ 0.001).

After the dialysis, the morphology of the U-251-MG cells on the hydrogels was observed after 3 and 7 div. The dyeing of the cytoskeleton, precisely the intermediate filaments GFAP, vimentin, and vinculin, as well as F-actin, which was marked by phalloidin, showed that after 3 div, the cells presented on all hydrogels an outspread morphology comparable to the control (Figure 8). The cells were relatively equally spread over the hydrogels in all conditions. After 7 div, the cells on the hydrogel with the highest TMAEA concentration and on the control were equally distributed over the area. The cells occurred in a stretched morphology, though the cells on the control indicated a significantly more elongated morphology than the cells on the 0.91 µmol/mg TMAEA hydrogel (Figure 8A–A‴,G–G‴,H–J; 0.91 µmol/mg TMAEA: area: 782.18 ± 34.96 µm^2^; circularity: 0.51 ± 0.02; length: 60.62 ± 2.51 µm; control: area: 699.62 ± 24.35 µm^2^; circularity: 0.32 ± 0.01; length: 74.86 ± 2.33 µm). The cells on the hydrogel with a low or medium TMAEA concentration, namely 0.06 µmol/mg, 0.11 µmol/mg, and 0.23 µmol/mg TMAEA, stretched sporadically but mostly the cells appeared in a round morphology, especially the cells on the hydrogels at 0.23 µmol/mg TMAEA and 0.11 µmol/mg TMAEA, which indicate significantly the most circular morphology (Figure 8C–C‴,D–D‴,E–E‴,H–J; 0.23 µmol/mg TMAEA: area: 549.89 ± 26.53 µm^2^; circularity: 0.67 ± 0.02; length: 43.9 ± 2.61 µm; 0.11 µmol/mg TMAEA: area: 482.37 ± 20.54 µm^2^; circularity: 0.68 ± 0.02; length: 39.41 ± 2 µm; 0.06 µmol/mg TMAEA: area: 669.5 ± 32.98 µm^2^; circularity: 0.57 ± 0.02; length: 49.97 ± 2.27 µm). Additionally, the cells tended to form clusters whereat sphere-like structures were rare. The hydrogel at 0.46 µmol/mg TMAEA showed an irregular cell distribution. While the cells were equally distributed in some areas, cell aggregates were formed in others. However, the evaluation revealed that the cells appeared in a significantly more stretched morphology than the cells on the low or medium charged hydrogels and comparable with the U-251-cells on the unfunctional hydrogel (Figure 8B–B‴,F–F‴,H–J; 0.46 µmol/mg TMAEA: area: 742.27 ± 31.2 µm^2^; circularity: 0.54 ± 0.02; length: 57.17 ± 2.48 µm; unfunctional: area: 765.09 ± 24.48 µm^2^; circularity: 0.43 ± 0.02; length: 62.87 ± 2.01 µm). By comparison of the dialyzed hydrogels with the undialyzed hydrogels, it was noticed that the cell spreading area and the length of the cells on the low and medium charged hydrogels were increased on the dialyzed hydrogels. On the other hand, the circularity of the cells was decreased on the dialyzed hydrogels. As with the comparison of the cell numbers, the effect was not seen on the hydrogel with the highest cationic concentration, underlining the thesis of the superior role of the cationic group compared to dialysis. A hydrogel with a high cationic concentration above a threshold of 0.46 µmol/mg TMAEA without dialysis results not only in a large total number of adherent cells but also in an outspread and healthy morphology comparable to the control. For hydrogels below this threshold, dialysis can restore the increased cell number and stretched morphology to a certain degree.

## 3. Conclusions

In this study, we evaluated a synthetic hydrogel system containing the cationic group TMAEA which is permanently positively charged and the concentration-dependent effect on the cultivation of U-251-MG cells. First, we can confirm a synergistic effect of the cationic group and the RGD motif on the adhesion of U-251-MG cells. The combination of both motifs in a hydrogel increased the number of adherent cells compared to hydrogels where only one motif was present. Furthermore, it was shown that the adhesion of cells was highly dependent on the concentration of TMAEA when undialyzed gels were used. Here, the highest number of living cells was observed on the hydrogels with the maximum concentration of 0.91 µmol/mg cationic moieties. Using lower concentrations of TMAEA resulted in a decrease of adherent cells.

Dialysis was used to remove any residual precursor polymer chains or solvents from the hydrogel. This purification step led to an overall increase of adherent cells for the unfunctional gel, and those with lower TMAEA concentration of 0.06 µmol/mg, 0.11 µmol/mg, and 0.23 µmol/mg. Only for the highest TMAEA concentration we could not observe any difference in cell adhesion with or without gel dialysis. We believe that this study provides valuable guidance for the preparation of fully synthetic and bioactive hydrogels for 2D cell experiments.

## 4. Materials and Methods

### 4.1. Materials for Hydrogel Synthesis

The chemicals used in this study were bought from the following suppliers and unless otherwise stated were used without further purification: PEG (average M_w_ = 3400) was purchased from Sigma Aldrich (Steinheim, Germany, now Merck KGaA). Acryloylchloride was purchased from Merck KGaA (Darmstadt, Germany). 4-Acryloylmorpholine (purity 97%), Iodmethane (purity 99%), and Triethylamine (purity 99%) were purchased from Acros Organics (Schwerte, Germany, now Thermo Fisher Scientific Inc.). Methacryloylchloride (purity 97%) was purchased from Alfa Aesar (Landau, Germany, now Thermo Fisher Scientific Inc.). Cystamine dihydrochloride (purity > 97%) was purchased from TCI (Tokyo, Japan). The initiator 2,2′-Azobis(4-methoxy-2,4-dimethylvaleronitrile) (V-70) was purchased from FUJIFILM Wako Chemicals Europe GmbH (Neuss, Germany). Dithiothreitol (purity 98%) was purchased from abcr GmbH (Karlsruhe, Germany). All solvents used were either HPLC-grade or technical grade. In the case of technical grade solvents, all solvents were distilled prior to use. Isopropanol, Dichloromethane, and Tetrahydrofuran were purchased from VWR (Bridgeport, NJ 08014, PA, USA). Dimethylsulfoxide (purity 99%) was purchased from Grüssing GmbH (Filsum, Germany).

### 4.2. Synthesis of TMAEA

The cationic monomer TMAEA was synthesized as previously reported by Salehi et al. [34]. Briefly, *N,N*-Dimethylaminoethylacrylate (DMAEA, 5.4 mL, 34.9 mmol, 1.0 eq.) was solved in THF (15 mL, 3 mL/g DMAEA) and cooled to 0 °C. Iodmethane (2.5 mL, 40.2 mmol, 1.15 eq.) in THF (15 mL, 3 mL/g DMAEA) was added dropwise to the stirring solution of DMAEA in THF. After complete addition of Iodmethane, the solution was left to stir at rt overnight. The precipitate was filtrated, washed with cold cyclohexane (2x), and dried in vacuum to yield a white crystalline solid (9.5 g, 95%).

### 4.3. Synthesis of BMAC

The bifunctional, disulfide containing monomer BMAC was synthesized as previously described by Zhang et al. [35]. Briefly, Cystamine dihydrochloride (5.0 g, 22.2 mmol, 1.0 eq.) and NaOH (3.55 g, 88.8 mmol, 4 eq.) were solved in Milli-Q^®^ water and cooled to 0 °C. Methacryloylchloride (4.3 mL, 44.41 mmol, 2 eq.) was added dropwise to the solution. The mixture was left to stir at rt for 3 h. The precipitate was filtrated, washed with water, and dried in vacuum. Recrystallisation from ethyl acetate yielded a white crystalline solid (8.4 g, 66%).

### 4.4. Prepolymer Synthesis

The prepolymers are synthesized via free radical polymerization and are hereinafter exemplary explained for the synthesis of the cationic polymer P2. The free radical polymerization was carried out in a 50 mL centrifuge tube sealed with a cap without any stirring. A solution of AMor (1.06 mL, 8.4 mmol, 14 eq.), BMAC (173 mg, 0.6 mmol, 1 eq.), and TMAEA (1711 mg, 6.0 mmol, 10 eq.), resulting in a composition of 56:4:40, in DMSO (8.9 mL, total concentration of all monomers is 1.5 mmol/mL) was prepared and purged with argon for 20 min. Afterwards, the initiator 2,2′-Azobis(4-methoxy-2,4-dimethylvaleronitrile) (V-70, 50 mg, 0.15 mmol, 0.25 eq.) was added to the purged solution and the centrifuge tube was placed in a drying chamber at 42 °C. After 30 min, the solution forms an in situ gel which is left in the drying chamber for 3 h to guarantee a good conversion of the monomers. The formed gel was then cut into small pieces with a scalpel and placed into a dialysis chamber for 2 days. The water was exchanged daily. The gel pieces were transferred to a 250 mL round-bottom flask and the disulfide bond of BMAC reduced by the addition of DTT (463 mg, 3 mmol, 5 eq. regarding the amount of BMAC) in a 1 M NaOH (1–2 mL) solution. The gel-DTT suspension was purged with argon until all gel pieces were dissolved (approximately 20–30 min). After filtration of unsolved impurities, the solution was reduced to a volume of about 20–30 mL and precipitated in slightly acidic Isopropanol (ratio of 20:1 excess) to collect the now soluble prepolymers.

For polymers P1 and P3, THF was used instead of DMSO for the polymerization.

### 4.5. Functionalization with RGD Peptide

For the functionalization with the peptide sequence MIC-6AHX-YGRGDS, a polymer with a composition of 92.4:7.6 (AMor:BMAC, 0.91 mmol/g thiol content) was used. About 3.6% functionalization (0.43 mmol/g) with the RGD peptide was desired. Therefore, the polymer (1 g) and the MIC-6AHX-YGRGDS peptide (421 mg, 0.44 mmol) were solved separately in 3 mL of Milli-Q^®^ water purged with argon. The pH-value of both solutions was set to 6.5–7.0 to prevent reoxidation of the thiols and the solutions were mixed. The resulting final solution was left to stir for 24 h at rt. Afterwards, the polymer was dialyzed against water for 1 day.

### 4.6. Synthesis of PEGDA3500

PEGDA3500 was synthesized as previously reported by Lee [36]. Briefly, PEG (M_w_ = 3400 g/mol, 10 g, 2.9 mmol, 1 eq.) and Triethylamine (804 µL, 5.8 mmol, 2 eq.) was solved in CH_2_Cl_2_ (75 mL, 7.5 mL/g PEG). Acryloyl chloride (959 µL, 11.6 mmol, 4 eq.) was slowly added dropwise to the solution and the final solution was left to stir overnight at rt. The organic phase was filtered and afterwards washed with saturated sodium bicarbonate. After drying with magnesium sulfate, the solution was precipitated in ice cold diethyl ether and dried in vacuum to yield a white crystalline solid (8.0 g, 78%).

### 4.7. Hydrogel Preparation

For the preparation of the hydrogels, stock solutions of the prepolymers and PEGDA3500 were used. For the preparation of stock solution, polymers and PEGDA3500 were solved in sterile 0.01 M PBS buffer and the pH adjusted to 6.8–7.4 with 1 M NaOH. For polymers a stock solution of 200 mg/mL and for PEGDA3500 a stock solution of 175 mg/mL were used. For the preparation of the gels, a Bio-Rad SDS-Page chamber with a gap of 1 mm was used. As an example, for a cationic hydrogel with a final cationic concentration of 0.911 µmol/mg, 1644 µL of the polymer stock solution was first mixed with 149 µL of sterile 0.01 M PBS buffer and then 1207 µL of PEGDA3500 stock solution was added, mixed, and then added to the gel cassette. After 2 h, the gel was removed from the cassette and samples were punched out of the gel with a Ø 13 mm cork stamp. 

### 4.8. NMR Spectroscopy

To analyze the polymer composition, ^1^H-NMR spectroscopy was used. Samples were analyzed on FT-NMR devices from Bruker (type Bruker Avance III HD Nanobay (400 MHz), Bruker Avance III HD (500, 600 and 700 MHz), and from Agilent (type Agilent Technologies DD2 (500 MHz). For the calculation of the polymer composition, the integrals of normalized integrals with respect to the methyl group of BMAC (3 protons of the methyl group of the methacrylate at 0.80–1.00 ppm), AMor (8 ring protons at 3.27–3.80 ppm), and TMAEA (9 methyl protons of the methyl groups of quaternary amine at 3.08–3.23 ppm) were used.

### 4.9. Ellman’s Assay

With Ellman’s assay, the thiol content of polymers can be determined as previously reported [27]. Briefly, stock solutions of polymers (1 mg/mL) and Ellman’s reagent DTNB (4 mg/mL) in 0.1 M phosphate buffer (pH 8) were prepared. For the sample solution, 1.25 mL of 0.1 M phosphate buffer (pH 8), 0.125 mL polymer stock solution, and 0.025 mL DTNB stock solution were mixed and stirred for 30 min. Afterwards, the absorbance of the yellow solution was measured at 412 nm with a VWR-UV6300 PC Double Beam Spectrophotometer from VWR. The thiol concentration was calculated by the law of Lambert–Beer using a molar attenuation coefficient of 14150 L mol^−1^ cm^−1^.

### 4.10. Rheology

For oscillatory measurements, a MCR 102 rheometer from Anton Paar was used. Samples were prepared in the same way as described in Section 4.4 except for filling of the solution in the gel cassettes. Instead, three samples of 500 µL were poured into 15 × 2 mm cylindrical molds and, to prevent evaporation, covered with parafilm. The samples were left overnight for full gelation and afterwards placed in water to enable complete swelling of the gels before performing oscillatory experiments. The experiments were carried out with a frequency of 1 Hz and a deformation of 1%.

### 4.11. Swelling Ratio

The swelling ratio (*Q_m_*) was calculated by comparison of the weight of a fully swollen gel with a dry gel. Therefore, the gels were weighed before oscillatory experiments and afterwards freeze-dried to remove the water. The swelling ratio can be calculated by the following equation:

$$Q_{m}=\frac{m_{s}-m_{d}}{m_{d}}$$

*m_s_*: weight of swollen gel, *m_d_*: weight of dry gel.

### 4.12. ζ-Potential

The ζ-potential was measured with a NanoBrook Zeta Potential Analyzer from ZetaPals. The samples were prepared in a 10 mM KCl solution with a final concentration of 1 mg/mL. The measurements were performed at 25 °C and in a set of 10 measurements.

### 4.13. REM Measurements

REM measurements were performed with a H-S4500 FEG device from Hitachi. For the measurements, dry samples were needed. Therefore, fully swollen hydrogels were freeze-dried to retain most of their initial shape. Right before the measurement, samples were frozen in liquid nitrogen and cut in half to measure the cross-section area of the gel during the experiment. Additionally, conductive modelling clay was used to increase the conductivity of the hydrogels.

### 4.14. U-251-MG Cell Culture

In this study, the human astrocytoma/glioblastoma cell lines U-251-MG (human glioblastoma cell line, WHO grade IV) was used [37,38]. The cells were stored in liquid nitrogen. To thaw the cells, an aliquot was warmed at 37 °C for 1 min. The cells were transferred into 37 °C warm medium (Minimum Essential Medium Eagle alpha (Sigma-Aldrich; Cat No.:M6199) supplemented with 10% *v*/*v* fetal calf serum (FCS; Thermo Fisher Scientific Inc.; Cat. No.: 10270-106) and 0.1% *v*/*v* gentamicin (Sigma-Aldrich; Cat. No.: G1397)) and centrifugalized for 5 min at 80 g (Multifuge 3 S-R; Heraeus; Hanau, Germany). Afterwards, the supernatant was discarded, and the pellet was resuspended in fresh medium. The cells were cultured in T-75 flasks (Greiner Bio-One; Cat. No.: 658175) at 37 °C and 6% *v*/*v* CO_2_. The medium was exchanged every 3–4 days and the cells were split once a week at a confluence of 80–90% in a ratio of 1:75. Therefore, the cells were washed once with phosphate buffered saline (PBS; 137 mM sodium chloride, 3 mM potassium chloride, 6.5 mM disodium hydrogen phosphate, 1.5 mM potassium dihydrogen phosphate; pH 7.4) and incubated with 1 mL 0.05% trypsin-EDTA (ethylenediaminetetraacetic acid; T/E; Thermo Fisher Scientific Inc.; Cat. No.: 25300-062) for 6 min at 37 °C and 6% *v*/*v* CO_2_. After trypsinization, the detachment of the cells was controlled by visual inspection using a microscope and the digestion was stopped by the addition of 5 mL medium. The cells were transferred into a 15 mL centrifuge tube (Sarstedt; Cat. No.: 62.554.502) and centrifugalized for 5 min at 80 g. The supernatant was discarded, and the pellet was resuspended in fresh medium. A total of 1:75 of the cell suspension was transferred into a T-75 flask with 15 mL warm medium and the cells were cultured at 37 °C and 6% *v*/*v* CO_2_.

For the analysis, hydrogels were placed in 4-well dishes (Thermo Fisher Scientific Inc.; Cat. No. 176740) or 24-well plates (Thermo Fisher Scientific Inc.; Cat. No. 142475). Undialyzed hydrogels were prepared with sterile ingredients, consequently additional sterilization was not necessary. Dialyzed hydrogels were sterilized by incubation in 70% ethanol for 1 h. Both hydrogels were washed thrice with PBS and were incubated with medium for 1 h at 37 °C and 6% *v*/*v* CO_2_ before cell plating. Cells were detached from the flasks using T/E and plated in a density of 40,000 cells per hydrogel. U-251-MG cells on hydrogels were cultured at 37 °C and 6% *v*/*v* CO_2_ for 3 and 7 days.

### 4.15. Live/Dead Staining

The live/dead staining was performed with the LIVE/DEAD^®^ Viability/Cytotoxicity Kit for mammalian cells (Thermo Fisher Scientific Inc.; Cat. No.: L3224). The cells were washed once with PBS and then incubated with the dye solution consisting of PBS supplemented with 4 μM EthD-1 and 2 μM calcein AM for 15 min at room temperature under exclusion of light. After the incubation, the cells were washed with PBS and fluorescence images were taken immediately (AxioZoom.V16; Zeiss; Oberkochen, Germany).

### 4.16. Immunocytochemistry

The cytoskeleton of the U-251-MG cells was visualized with an immunocytochemical staining. The cells were fixed with 4% *w*/*v* paraformaldehyde (Carl Roth GmbH & Co. KG; Cat. No.:4235.1) and washed thrice with PBT-1 [PBS supplemented with 1% *w*/*v* bovine serum albumin (BSA; Carl Roth GmbH & Co. KG; Cat. No.:8076.2) and 0.1% *v*/*v* Triton-X-100 (AppliChem GmbH; Cat. No.: A4975,0500)]. Primary antibodies were diluted in PBT-1, namely polyclonal anti-GFAP antibody (1:300; rabbit; Agilent DAKO; Cat. No.: Z0334; RRID: AB_100 13382), monoclonal anti-vimentin antibody (1:300; mouse; Sigma-Aldrich; Cat. No.: V2258; RRID: AB_261856), and monoclonal anti-vinculin antibody (1:200; mouse; Sigma-Aldrich; Cat. No.: V9131; RRID: AB_477629). Additionally, phalloidin-atto 488 (Sigma-Aldrich; Cat. No.: 49409) was used for the labeling of F-actin. The cells were incubated with the primary antibody and phalloidin solution for 1 h at room temperature under exclusion of light on a hinged plate. Afterwards, the cells were washed three times with PBS/A (PBS supplemented with 0.1% *w*/*v* BSA). The secondary antibodies were diluted in PBS/A, namely polyclonal anti-mouse antibody (1:300; Cy3; anti mouse IgG/IgM; goat; Jackson ImmunoResearch Labs; Cat. No.: 115-165-068; RRID: AB_ 2338686) and polyclonal anti-rabbit antibody (1:300; AlexaFlour 488; anti rabbit IgG; goat; Jackson ImmunoResearch Labs; Cat. No.: 111-545-045; RRID: AB_2338049). The nuclei were stained with bisbenzimide (Hoechst 33258, 1:100.000, Sigma-Aldrich; Cat. No.: 94403). The incubation with the secondary antibody and nuclei marker solution took 1 h at room temperature under exclusion of light on a hinged plate. Subsequently, the cells were washed with PBS and mounted with 50% *v*/*v* PBS and 50% *v*/*v* glycerol (Fisher Chemicals; Cat. No.: G/0650/15) for microscopy. Images were taken with the AxioZoom (AxioZoom.V16; Zeiss; Oberkochen, Germany).

### 4.17. Quantification and Statistics

The live/dead staining was quantified with the cell counter plug-in by ImageJ (Version 1.53q). The cell spreading area, length, and circularity were measured with the same program. The statistical evaluation was performed with *GraphPad Prism* (Version 5.02). The data sets were analyzed regarding their normality distribution using the Shapiro–Wilk test. Data sets which were not normally distributed were analyzed with a Kruskal–Wallis test and the *post hoc* Dunn’s test to determine their *p*-value. To identify the level of significance of two different groups, a two-way ANOVA and the *post hoc* Bonferroni test were used. The following *p*-values were defined as significant: * *p* ≤ 0.05, ** *p* ≤ 0.01, and *** *p* ≤ 0.001. Cell experiments were performed in biological triplicates (N = 3), except the pilot study. For the evaluation of the cell number, eight pictures per experiment and condition were taken and quantified (n = 24). For the evaluation of the cell spreading area, length, and circularity, five cells per image were measured (n = 120). The circularity was defined as follows:

$$circularity=4\pi *\left(\frac{area}{perimeter^{2}}\right)$$

A relative value of 1.0 indicates a perfect circle, whereas a value of 0.0 represents an elongated object.

## Figures and Tables

**Table 1:** Polymer composition and their respective analytical data.

Polymer	Composition AMor:BMAC:TMAEA:RGD [mol%]		BMAC Content Ellman’s Assay [mol%]	SEC [g/mol] ^(a)^	*Ð*
	Theoretical	^1^H-NMR Spectroscopy			
P1	96:4:0:0	95.6:4.4:0:0	3.1	25,000	1.54
P2	56:4:40:0	59.8:2.4:37.9:0	3.4	- ^(b)^	- ^(b)^
P3	88:4:0:8	88.0:2.7:0:9.3	2.7	33,200	2.7

^(a)^ Samples solved in DMF + 5 g/L LiBr, ^(b)^ no data due to insolubility in DMF.

**Table 2:** Physical data of hydrogels for 2D cell experiments.

Gel	Composition	Concentration of Functional Groups [µmol/mg] ^(c)^	Q ^(d)^	E ^(e)^ [kPa]	ζ-Potential ^(f)^ [mV]
G1	AMor-BMAC	-	10.3 ± 0.1	7.7 ± 0.4	−3.9 ± 1.6
G2 ^(a)^	AMor-BMAC-TMAEA	0.91	11.1 ± 0.4	8.8 ± 1.4	+42.1 ± 3.7
G3	AMor-BMAC-RGD	0.26	14.7 ± 0.1	1.7 ± 0.2 ^(g)^	−15.4 ± 1.8
G4 ^(b)^	AMor-BMAC-TMAEA-RGD	0.63/0.13	14.4 ± 0.1	2.1 ± 0.3 ^(g)^	+36.6 ± 2.5
G5 ^(a)^	AMor-BMAC-TMAEA	0.46	10.0 ± 0.2	9.1 ± 0.7	+35.1 ± 2.8
G6 ^(a)^	AMor-BMAC-TMAEA	0.23	10.0 ± 0.1	9.6 ± 0.8	+31.5 ± 1.9
G7 ^(a)^	AMor-BMAC-TMAEA	0.11	10.2 ± 0.2	8.6 ± 1.1	+24.9 ± 3.3
G8 ^(a)^	AMor-BMAC-TMAEA	0.06	9.6 ± 0.2	11.1 ± 0.4	+20.0 ± 1.2

^(a)^ Precise cationic concentrations obtained by mixing **P2** with **P1**, ^(b)^ gel obtained by mixing **P2** and **P3** in a 50:50 ratio, ^(c)^ concentration of the functional groups in µmol per mg gel, ^(d)^ measured in triplicates in PBS buffer, dry weight obtained by freeze-drying fully swollen gels, ^(e)^ rheology measurements of fully swollen gels in PBS buffer performed in triplicates with a frequency of 1 Hz and a deformation of 1%, ^(f)^ measured in a 1 mM KCl solution with a concentration of 1 mg/mL of free polymer at pH = 7–8 and 25 °C. Mean value derived from 10 measurements. For different TMAEA concentrations, a mixed solution of P1 and P2 with a final polymer concentration of 1 mg/mL is used, ^(g)^ RGD gels were formed in 10 wt% solutions while other gels were formed in 18 wt% solutions.

## Data Availability

We provide Supplementary Materials that will be published with the manuscript.

## Supplementary Materials

The following supporting information can be downloaded at: https://www.mdpi.com/article/10.3390/gels8120827/s1, Figure S1: ^1^H-NMR spectrum of TMAEA in D_2_O. Figure S2: ^1^H-NMR spectrum of BMAC in CDCl_3_. Figure S3: ^1^H-NMR spectrum of PEGDA3500 in CDCl_3_. Figure S4: Exemplary ^1^H-NMR spectrum of PAMor-co-BMAC P1 in D_2_O. Figure S5: Exemplary ^1^H-NMR spectrum of PAMor-co-BMAC-co-TMAEA P2 in D_2_O. Figure S6: Exemplary ^1^H-NMR spectrum of PAMor-co-BMAC-co-RGD P3 in D_2_O. Table S1. Physical data of hydrogels for 2D cell experiments in water.
