# Supplementary material for: Concentration Dependent Effect of Quaternary Amines on the Adhesion of U251-MG Cells
Source: Gels. 2022 Dec 15;8(12):827. doi: 10.3390/gels8120827 (PMC9777631; doi:10.3390/gels8120827)
Supplement: Supplementary file 1 [file gels-08-00827-s001.zip › gels-2078041-supplementary.pdf]

gels

MDPI

Supplementary

Concentration Dependent Effect of Quaternary Amines on the Adhesion of U251-MG Cells

Nils Stamm [1], Kristin Glotzbach [2], AndreasFaissner [2,*] and Ralf Weberskirch [1,*]

[1] Faculty of Chemistry and Chemical Biology, TU Dortmund University, 44227 Dortmund, Germany
[2] Department of Cell Morphology and Molecular Neurobiology, Ruhr Universität Bochum, 44801 Bochum, Germany
* Correspondence: andreas.faissner@rub.de (A.F.); ralf.weberskirch@tu-dortmund.de (R.W.); Tel.: +49-234-32-28851 (A.F.); +49-231-755-3863 (R.W.)

Supplementary Data

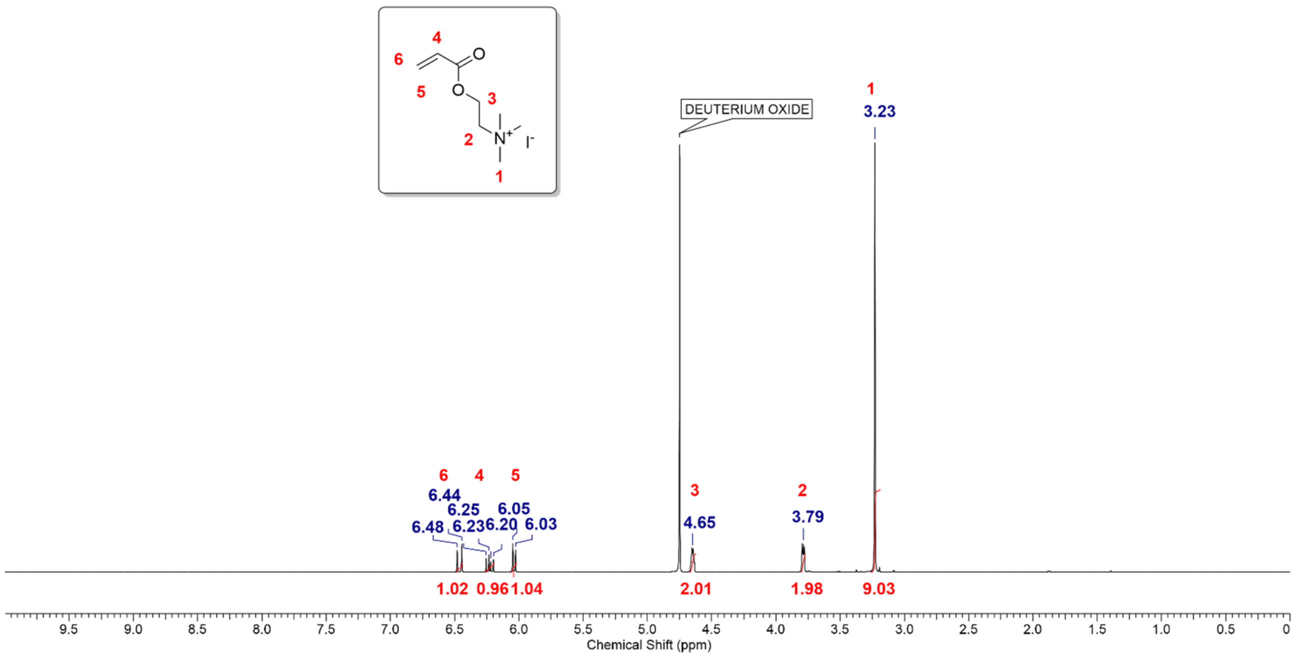

Figure S1. ^{1}H-NMR spectrum of TMAEA in D_2O.

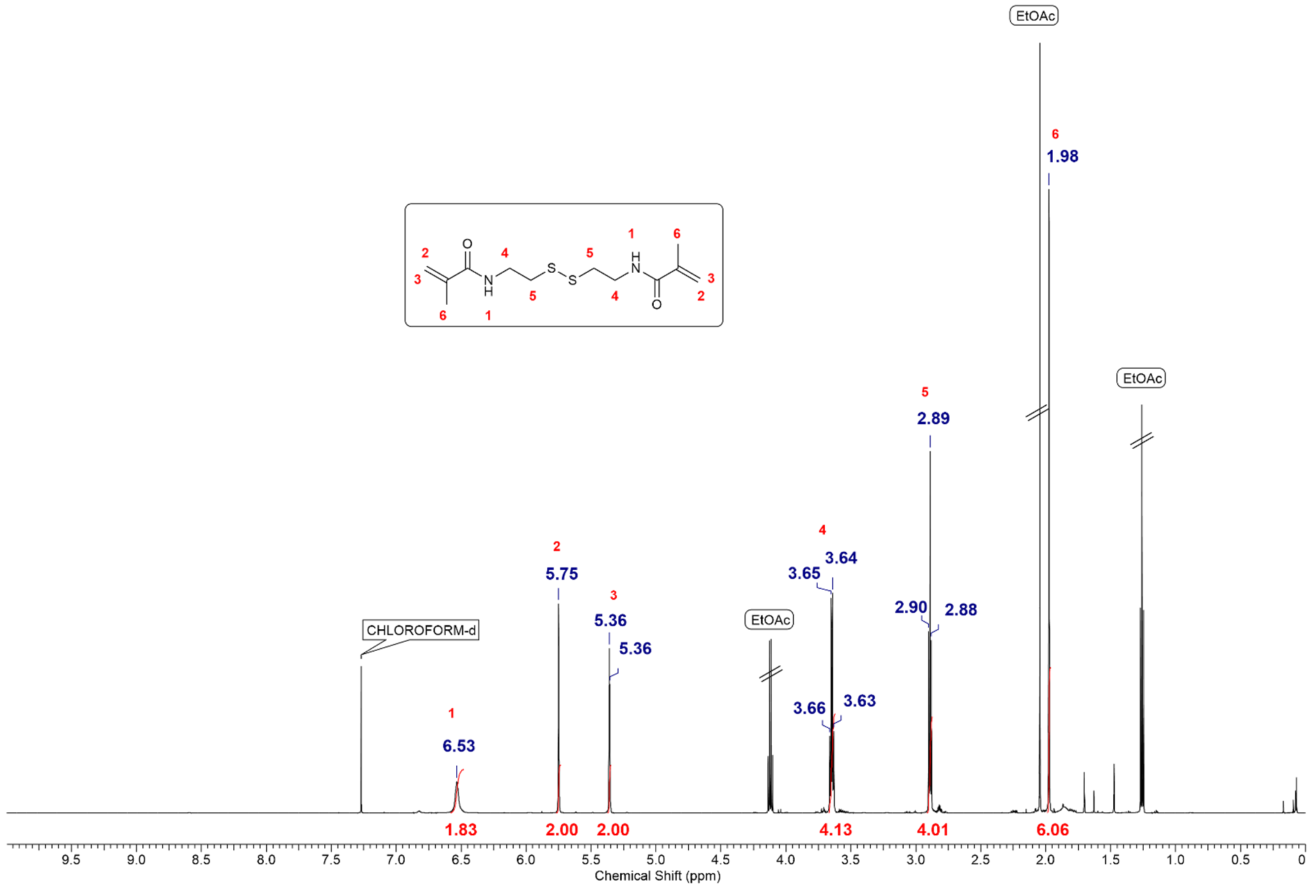

Figure S2. ^{1}H-NMR spectrum of BMAC in $CDCl_3$.

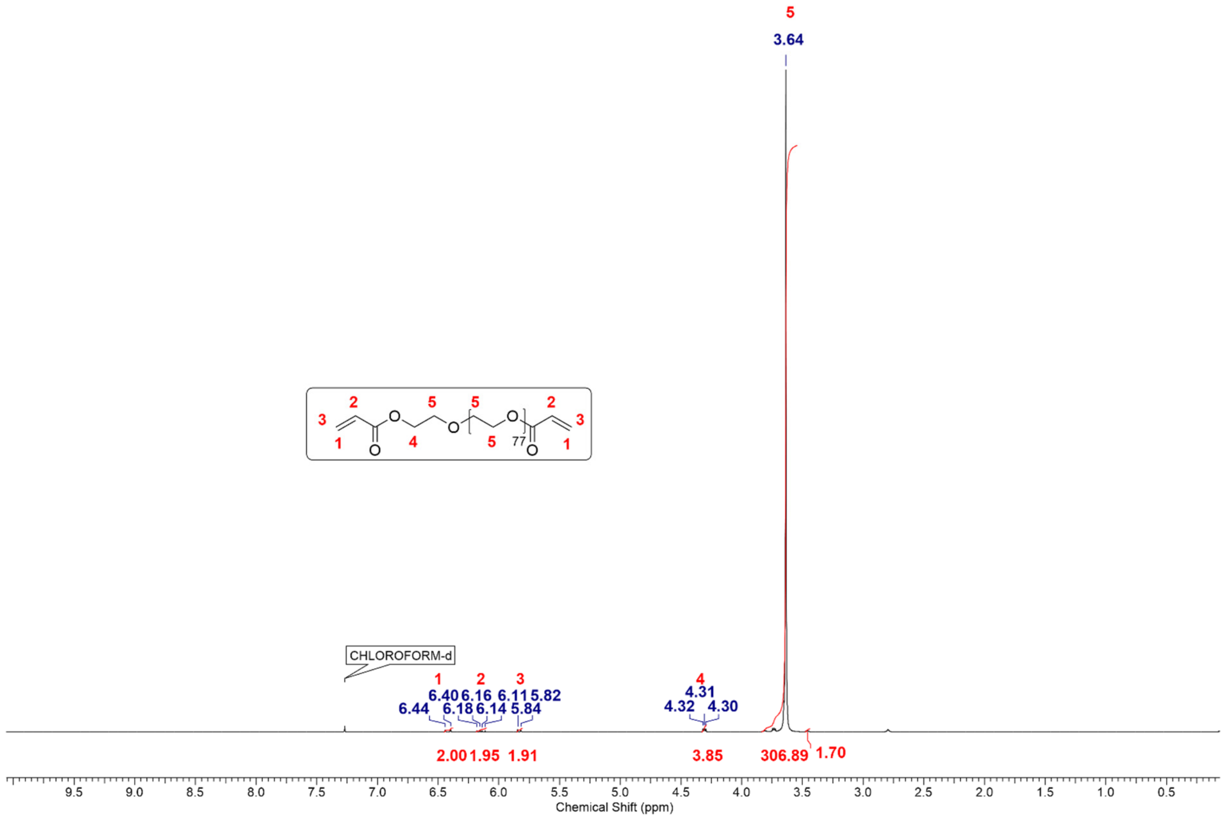

Figure S3. ^{1}H-NMR spectrum of PEGDA3500 in $CDCl_3$.

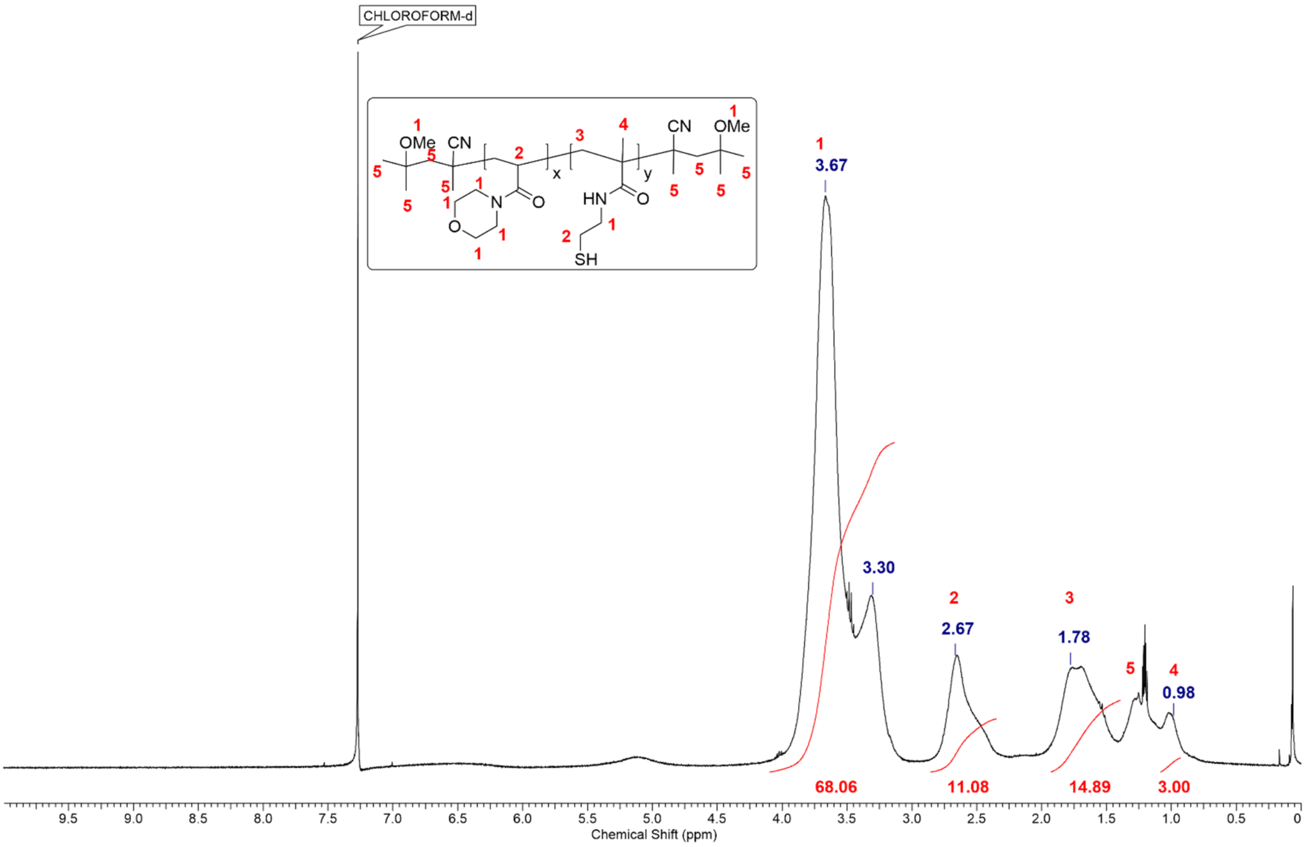

Figure S4. Exemplary ^{1}H-NMR spectrum of PAMor-co-BMAC **P1** in D_2O.

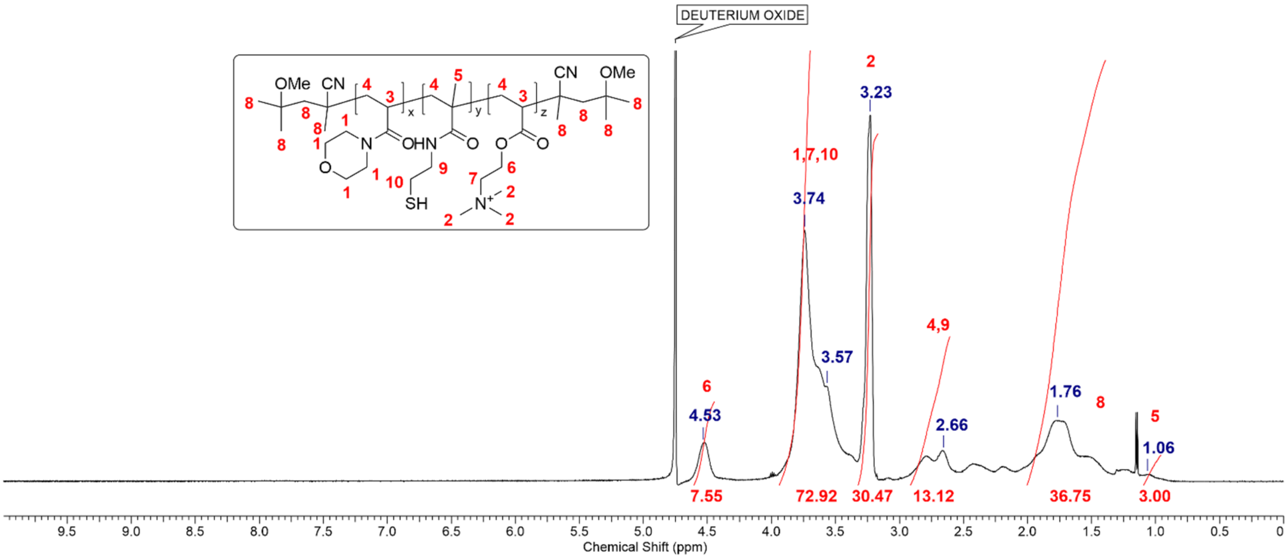

Figure S5. Exemplary ^{1}H-NMR spectrum of PAMor-co-BMAC-co-TMAEA **P2** in D_2O.

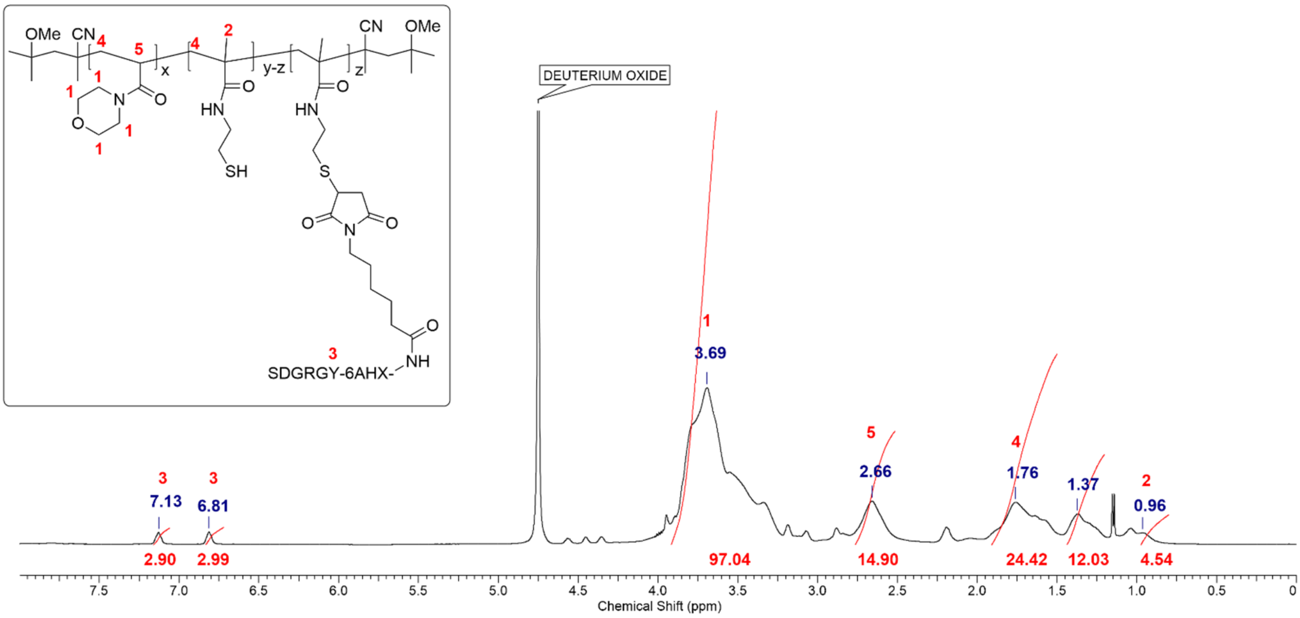

Figure S6. Exemplary ^{1}H-NMR spectrum of PAMor-co-BMAC-co-RGD **P3** in D_2O.

Table S1. Physical data of hydrogels for 2D cell experiments in water.

Gel	Composition	Concentration of Functional Groups [µmol/mg][c]	*Q* [d]	*E* [e] [kPa]	ζ-Potential [f] [mV]
G1	AMor-BMAC	-	13.3±0.2	7.8±0.2	-3.9±1.6
G2[a]	AMor-BMAC-TMAEA	0.91	16.9±0.2	15.4±3.1	+42.1±3.7
G3	AMor-BMAC-RGD	0.26	34.1	2.2[g]	-15.4±1.8
G4[b]	AMor-BMAC-TMAEA-RGD	0.63/0.13	31.4	4.7[g]	+36.6±2.5
G5[a]	AMor-BMAC-TMAEA	0.46	12.4±0.3	14.2±3.0	+35.1±2.8
G6[a]	AMor-BMAC-TMAEA	0.23	9.5±0.1	10.6±1.3	+31.5±1.9
G7[a]	AMor-BMAC-TMAEA	0.11	9.0±0.4	10.6±2.5	+24.9±3.3
G8[a]	AMor-BMAC-TMAEA	0.06	8.6±0.1	10.8±1.4	+20.0±1.2

a) Precise cationic concentrations obtained by mixing **P2** with **P1**, b) gel obtained by mixing **P2** and **P3** in a 50:50 ratio, c) concentration of the functional groups in µmol per mg gel, d) measured in triplicates in water, dry weight obtained by freeze-drying fully swollen gels, e) rheology measurements of fully swollen gels in water done in triplicates with a frequency of 1 Hz and a deformation of 1%, f) measured in a 1 mM KCl solution with a concentration of 1 mg/mL of free polymer at pH=7-8 and 25 °C. Mean value derived from 10 measurements. For different TMAEA concentrations a mixed solution of P1 and P2 with a final polymer concentration of 1 mg/mL is used, g) RGD gels were formed in 10wt% solutions while other gels were formed in 18wt% solutions.
